# Sensorimotor Theta Oscillations Coordinate Speech Movements

**DOI:** 10.1101/2025.10.09.681482

**Published:** 2025-10-10

**Authors:** Yitzhak Norman, Loren M. Frank, Edward F. Chang

**Affiliations:** 1 Department of Neurological Surgery, University of California, San Francisco; San Francisco, CA 94143, USA.; 2 Weill Institute for Neuroscience, University of California, San Francisco; San Francisco, CA 94143, USA.; 3 Graduate Program in Bioengineering, University of California, Berkeley–University of California, San Francisco; Berkeley, CA, USA.; 4 Howard Hughes Medical Institute, University of California, San Francisco; San Francisco, CA 94143, USA.; 5 Department of Physiology and Psychiatry, University of California, San Francisco; San Francisco, CA 94143, USA.; 6 Kavli Institute for Fundamental Neuroscience, University of California, San Francisco; San Francisco, CA 94158 USA.

## Abstract

Fluent speech depends on precisely timed motor commands that coordinate rapid transitions between successive articulatory gestures. Using direct cortical recordings, we identified a prominent sensorimotor theta oscillation (6–10 Hz) that supports this coordination. During articulation, premotor speech circuits exhibited enhanced theta phase coherence, with elevated population activity near theta troughs. The oscillation’s frequency remained remarkably stable across varying speech rates and cognitive states, consistent with an intrinsically generated rhythm. Vocal-tract kinematics revealed pulse-like movements at 6–10 Hz, tightly coupled to cortical theta phase. At a mesoscopic scale, theta cycles structured sequential sensorimotor activations encoding articulatory gestures, with syllable identity optimally decodable following theta troughs. These findings identify theta oscillations as an intrinsic timing mechanism that coordinates the distributed and synergistic motor control underlying fluent speech.

Speech is a defining human behavior, enabling us to express an unlimited range of thoughts by flexibly combining a limited set of basic phonetic elements. To speak fluently, the brain must generate rapid and precisely timed motor commands—at the scale of tens of milliseconds (1)—that coordinate the activity of nearly 100 muscles within the vocal tract (2). Each speech sound is produced through coordinated, synergistic movements of the tongue, lips, jaw, and other articulators, which dynamically shape specific constriction gestures (3). In fluent speech, transitions between these gestures are swift and seamless.

While previous research has shown that distributed neural activations within the ventral sensorimotor cortex (vSMC) play a central role in driving articulatory gestures (1, 4–10), the mechanism that ensures precise temporal coordination among the underlying neuronal populations remains enigmatic. Specifically, how does the brain endogenously activate these distributed neuronal representations in unison at precisely the right moment? How does it orchestrate successive motor commands to enable smooth transitions from one constriction gesture to the next? Uncovering this coordination mechanism is essential not only for deepening our understanding of motor control circuits underlying speech and other dexterous behaviors in the human brain, but also for advancing novel therapeutic strategies for speech and motor disorders and improving the precision of brain-computer interface technologies (11).

Insights from animal models point to a potential coordination mechanism rooted in intrinsic neural oscillations. In primates and rodents, rhythmic activity—particularly in the 6–10 Hz theta/alpha range—has been shown to synchronize neuronal spiking and population-level processing within and across brain regions (12–19). Across species, such oscillations have been implicated in the temporal coordination of diverse motor behaviors (20–24), including non-human primate vocalization (25) and human reading saccades (26). In the rodent hippocampus, theta oscillations organize information flow (27–29) and align hippocampal activity with locomotor (30), olfactory (31, 32), somatosensory (33, 34) and prefrontal representations on timescales of ~20–50 milliseconds (13, 15, 35). Together, these findings suggest that intrinsic oscillatory dynamics within the theta range can facilitate temporally precise interactions among spatially dispersed neuronal populations.

In humans, converging evidence links theta activity to large-scale interregional communication (36, 37), most notably during spatial navigation (38–42) and medial temporal lobe memory processing (43–45). Yet whether theta oscillations organize the processing of specific information streams remains unclear. Here, we tested whether theta rhythmicity supports the coordination of distributed motor control in the human brain—specifically, the motor control of speech, which relies on extensive premotor networks to orchestrate the rapid and complex muscle synergies underlying articulatory gestures (5, 10, 46).

Using high-density electrocorticographic (ECoG) recordings in neurosurgical patients, we identify a robust 6–10 Hz theta oscillation in the local field potentials (LFPs) of the vSMC and adjacent regions. This intrinsic neural rhythm exhibits stable power and frequency during ongoing articulation and shows coherent phase across widely distributed, speech-responsive sites. Crucially, during fluent speech, both vocal tract kinematics and cortical representations of articulatory movements are tightly coupled to specific phases of the oscillation. Taken together, our findings suggest that this circuit-level theta rhythm functions as an intrinsic timing mechanism—a shared rhythmic scaffold—that synchronizes premotor neuronal populations to coordinate the rapid and precisely timed articulatory movements required for fluent speech.

## Results

**Fig. 1 |:**
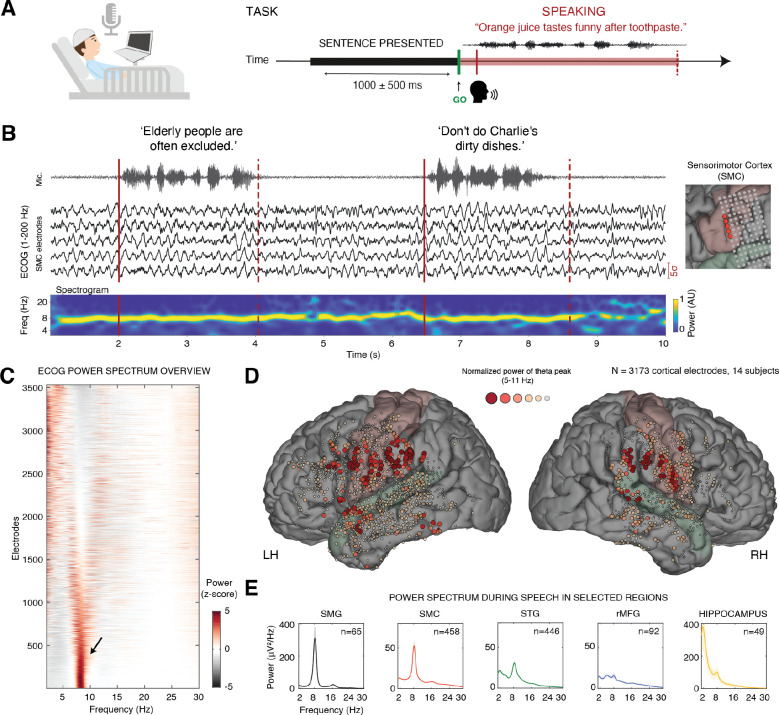
Prominent 8 Hz neural oscillation centered in the sensorimotor cortex during speech production. **(A)** Intracranial electrodes were implanted in the cortex and hippocampus as part of neurosurgical treatment for medically intractable epilepsy. During the task, participants spoke hundreds of English sentences covering the full phonetic inventory. **(B)** Example of 8 Hz theta oscillation during speech production, as it appears in five speech-responsive sensorimotor sites in one representative patient (wideband ECOG signal, filtered between 1–200Hz; right panel shows electrode location). The spectrogram at the bottom shows peak frequency around 8 Hz. This ongoing rhythm was detectable also during inter-trial silence period, pointing to its ongoing, intrinsic nature. Voice amplitude is shown at the top. **(C)** An overview of the power spectral density during speech production across 3,599 recording sites in 14 patients. A prominent power concentration in the high theta range (6–10 Hz) is evident across a major portion of the electrodes, with 41% of them displaying a single, distinct, theta peak (black arrow). **(D)** Cortical electrodes were color-coded according to the magnitude of their theta-band spectral peak. An evident cluster is observable over the sensorimotor cortex (SMC; painted in light pink) and surrounding areas, including the supramarginal gyrus (SMG), inferior frontal gyrus (IFG) and superior temporal gyrus (STG; painted in pale green). **(E)** Mean power spectrum during speech across selected regions of interest. From left to right: speech-responsive SMG sites (black, n=65), speech-responsive SMC sites (red, n=458), speech-responsive STG sites (green, n=446), speech non-responsive sites in rostral MFG (blue, n=92), and hippocampal electrodes (yellow, n=49). Notably, a distinct and robust 8 Hz spectral peak is observed in the SMC, SMG, and STG—cortical regions involved in speech motor control. A similar spectral peak is also evident in the hippocampus, indicating spatial propagation of this oscillation into subcortical structures. As a control region, non-responsive electrodes in the rostral MFG do not exhibit this prominent 8 Hz peak. Shaded areas represent least squares mean estimates ± SE across electrodes, derived from a linear mixed-effects model.

High-density electrocorticographic (ECoG) recordings were obtained from 17 patients undergoing intracranial epilepsy monitoring (5 females, 12 males, age range: 22–60, M=36.3). Participants spoke hundreds of sentences from the MOCHA-TIMIT corpus (47), covering the full range of the English phonetic inventory. Sentences were displayed on a screen, and after receiving a go cue, participants read them aloud at their natural speaking rate (Fig. 1A and Methods for task details). In addition, participants completed control tasks that included spontaneous speech in natural conversation, passive listening to sentences, and silent rest.

A total of 3,599 electrodes were analyzed, of which 1,438 were identified as speech-responsive sites—defined as sites exhibiting a significant increase in high-gamma (HG) amplitude during reading aloud compared to either pre- or post-utterance silent periods (P < 0.05, FDR corrected, see Fig. S1 and Methods). During articulation, we observed a prominent LFP oscillation in speech-responsive sensorimotor areas and adjacent regions. This oscillation was robust and widespread, persisted throughout the articulatory process, and could often be detected during inter-trial silence periods—consistent with an ongoing, intrinsically generated rhythm (Fig. 1B).

To characterize the spectral signature of this oscillation across recording sites, we computed the power spectral density for each electrode within a time window of −1 to 5 sec relative to speech onset (see Methods). Fig. 1C summarizes the resulting spectra, focusing on frequencies below 30 Hz. We observed a distinct theta-band peak centered around 8 Hz (black arrow), rising above the 1/f background, was observed in 41% of all cortical electrodes—defined by a peak prominence > 3σ relative to the z-scored, 1/f-corrected power spectrum (see Methods). Additional electrodes exhibited a broader, yet still pronounced, power concentration within the high-theta/lower-alpha range (6–10 Hz). Fig. 1D shows the anatomical locations of these electrodes, color-coded by the magnitude of the detected spectral peak. The oscillation was particularly prominent in the vSMC (see Fig. S1 for example electrodes) and neighboring regions.

To assess whether the spatial distribution of this rhythm aligns with speech production circuits, we computed the mean power spectrum across speech-responsive electrodes in key regions of interest (Fig. 1E). This analysis revealed a narrow-band spectral peak centered around 8 Hz during articulation, consistently observed across multiple nodes of the speech production network, including the vSMC, supramarginal gyrus (SMG), superior temporal gyrus (STG), and more distal structures such as the hippocampus. To further assess functional specificity, we compared theta power during speech between vSMC electrodes and non-responsive electrodes in a nearby cortical region outside the speech production network—specifically, the rostral middle frontal gyrus (rMFG). This within-subject comparison revealed significantly stronger theta power in vSMC (6–10 Hz power, vSMC vs. rMFG: t(548) = 2.63, P = 0.009; mixed-effects analysis, see Methods).

We hypothesize that the prominent vSMC-centered oscillation observed during speech serves as an intrinsic timing mechanism—a circuit-level “metronome”—that provides a rhythmic scaffold for synchronizing distributed premotor populations controlling speech movements. This synchronization is essential for producing the precisely timed vocal-tract gestures that underlie fluent and continuous speech.

This hypothesis leads to several key predictions, which we examine in the following sections: (1) *Frequency stability:* to provide a reliable rhythmic reference, the oscillation should maintain a stable frequency during articulation. (2) *Phase coherence:* to coordinate a shared central rhythm, theta phase should be coherent across spatially dispersed speech-production sites. (3) *Activity modulation:* local population firing should exhibit significant modulation by theta phase. (4) *Behavioral correlation:* vocal tract kinematics are expected to show transient movements phase-locked to theta. (5) *Mesoscopic-level orchestration*: patterns of distributed premotor activations, encoding specific articulatory gestures, should be spatiotemporally organized by the theta cycle.

### Intrinsic stability of the sensorimotor theta rhythm

The first prediction of our intrinsic scaffold hypothesis is that the oscillation’s central frequency should remain stable despite moment-to-moment fluctuations in speech syllable rate—providing a consistent temporal reference that can structure the behavior rather than being passively driven by it.

**Fig. 2.**
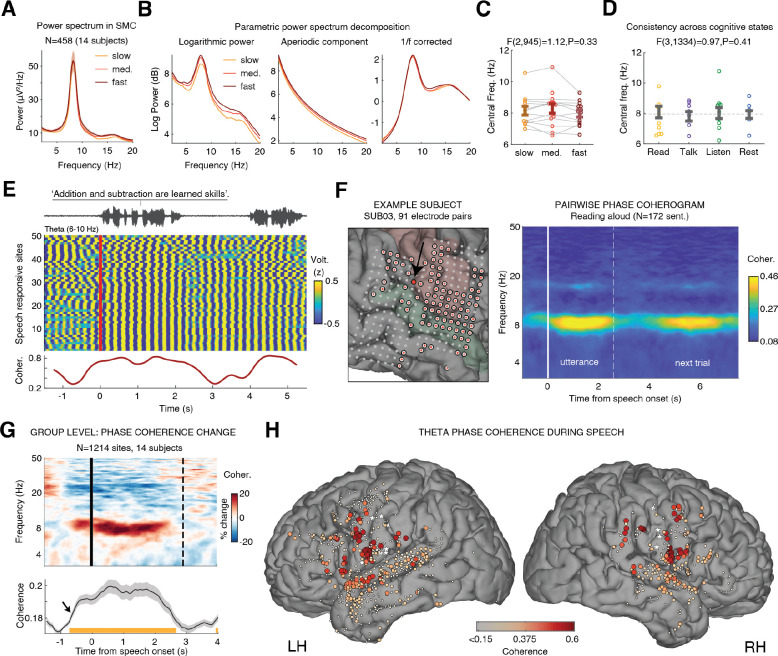
Intrinsic oscillatory stability and inter-regional theta phase coherence during speech. **(A)** Power spectrum during slow (bottom third), medium (middle third), and fast (top third) utterances. Despite substantial variation in syllable rate (2.9, 3.8, and 4.7 syl/s, respectively), the power spectrum remained markedly stable, peaking consistently around 8 Hz. **(B)** Parametric decomposition of the power spectrum in SMC sites (53) into aperiodic (1/f) and oscillatory components revealed a clear oscillatory peak in the high theta range (6–10 Hz). **(C)** The central frequency of theta-range peaks did not differ significantly across speech rates (F(2,890)=1.61, P=0.20, mixed-effects analysis; grand average: 8.2 ± 0.16 Hz). (D) The oscillation’s central frequency likewise remained stable across behavioral states—reading aloud, spontaneous speech, passive listening, and silent rest (F(3,1244)=0.36, P=0.78, mixed-effects analysis). **(E)** Example from a representative patient showing increased inter-site theta phase coherence during articulation. Top: voice amplitude. Bottom: sliding-window phase coherence (0.5-s windows, 0.05-s step) across 50 speech-responsive sites. **(F)** Averaged pairwise coherograms between a selected vSMC site exhibiting a prominent theta peak (black arrow) and all other speech-responsive sites in a representative patient (91 electrode pairs, marked with red dots; N = 172 sentences). A selective increase in phase coherence is evident in the 6–10 Hz range during articulation (dashed vertical line marks the median utterance duration). **(G)** Top: Group-level analysis showing the grand average change in phase coherence during articulation, computed across all speech-responsive sites relative to a selected vSMC site in each patient that exhibited a prominent theta oscillation. Bottom: phase coherence in the 6–10 Hz range increased significantly relative to the pre-speech silent period [−1 to 0] (timepoint-by-timepoint mixed-effects analysis, P < 0.05, FDR-corrected). Note the preparatory increase in coherence preceding speech onset (black arrow). Shaded area indicates the SE of the mixed-effects model estimates. **(H)** Anatomical distribution of theta phase coherence among speech-responsive electrodes during articulation (1,214 pairs, 14 subjects). White stars mark the selected theta sites used as reference for the pairwise coherence computation.

To test this, we pooled fluently articulated sentences within each subject (excluding those with speech errors or repairs) and grouped utterances into slow/medium/fast tertiles based on syllable rate (mean ± SD: slow, 2.9±0.5; medium, 3.8±0.5; fast, 4.7±0.6 syl/s). Power spectra from speech-responsive SMC sites were computed separately for each tertile (Fig. 2A). We then applied parametric spectral decomposition using the FOOOF algorithm (48) to separate the aperiodic 1/f component from the oscillatory components (see Methods). Across rates, a robust peak was consistently present around 8 Hz (Fig. 2B). The central frequency of peaks within the broader theta range (5–11Hz) did not vary with speech rate (mixed effects: F(2,1265) = 1.66, P = 0.19; Fig. 2C). This stability, despite substantial variation in syllable rate, supports the interpretation that this oscillation reflects an intrinsic, self-organized rhythm rather than a passive byproduct of articulation or its acoustic consequences; were it a passive byproduct, the frequency would be expected to track speaking rate.

A silent-miming control in five subjects further ruled out acoustic feedback as the source of the oscillation: the oscillation was evident during both speaking aloud and silent miming (articulatory movements performed without sound), with no significant changes in central frequency, peak width or height (Fig. S2A-E).

Participants also completed control tasks, including spontaneous speech production (autobiographical storytelling), passive listening, and silent rest (see Methods). Applying the FOOOF algorithm to power spectra from SMC/SMG sites during these tasks showed that the central frequency of the sensorimotor oscillation did not differ significantly across behavioral states, further supporting its intrinsic origin (Fig. 2D; see Fig. S3 for more detail). Notably, relative to both passive listening and silent rest recorded outside the task, during articulation the oscillation showed a significantly narrower spectral peak—reflecting reduced frequency variability (mixed effects analysis: speaking-vs-listening: F(1,558)=4.51, P = 0.02; speaking-vs-silence: F(1,406)=7.68, P = 0.006; Fig. S3). This suggests that the rhythm becomes more periodic during articulation and relaxes during motor idling states.

Previous studies that investigated sensorimotor oscillations during other (more discrete) motor behaviors have reported mixed patterns of event-related synchronization and desynchronization (aka, “mu suppression”) time-locked to movement onset (49–52). To determine whether the oscillation observed here showed a more transient event-related power modulation relative to speech onset, we computed peri-utterance wavelet spectrograms in SMC and SMG electrodes (Fig. S4; see Methods). We analyzed the spectrograms using two complementary normalization techniques: relative power, in which power at each frequency is normalized by the total power across all frequencies—highlighting the relative shape of the spectrum at each time point; and baseline normalization, showing power changes relative to the immediate pre-speech silent interval.

Both analyses underscored the intrinsic stability of the oscillation. Relative power revealed sustained and focal theta-band activity throughout articulation, whereas baseline-normalized power showed minimal speech-related modulation—a stability that stands out against the pronounced power reductions observed in neighboring mu/beta frequencies. Quantifying the temporal profile of power modulation within a narrow band around the SMC’s central theta frequency (8.2 ± 1 Hz) revealed no significant change during articulation (min P = 0.15; timepoint-by-timepoint mixed-effects analysis vs. pre-speech baseline [–1 to 0], FDR-corrected). In contrast, adjacent frequencies—particularly in the higher mu range (11–13 Hz)—exhibited robust and significant power reductions during articulation (P < 0.05, FDR-corrected; Fig. S4).

Together, these spectral analyses point to an intrinsically-generated theta rhythm that remains stable across diverse speech behaviors and becomes more spectrally focused during articulation, making it well suited to serve as a reliable rhythmic coordinator for continuous speech.

### Speech-triggered increase in theta phase coherence

Having confirmed power and frequency stability, we next examined whether the *relative phases* across sites were also consistent with this oscillation functioning as a shared temporal scaffold for premotor neuronal coordination. We filtered the raw ECoG signal within the theta range (6–10 Hz) and time-locked it to speech onset. Figure 2E shows the theta-band voltage traces from 50 speech-responsive electrodes recorded simultaneously during one sentence in a single subject. During articulation, the ongoing voltage fluctuations exhibited rhythmic alignment across sites—indicative of large-scale, circuit-wide phase synchronization.

To quantify this phenomenon, we computed inter-electrode phase coherence (i.e., peri-speech coherograms) across 3–50 Hz frequencies, separately for each subject. The analysis was performed pairwise, comparing each speech-responsive electrode to a reference vSMC electrode exhibiting a prominent theta peak (Fig. 2F; see Methods). To minimize volume conduction effects, neighboring channels (<15 mm apart) were excluded from the analysis (Fig. S5).

This revealed a significant increase in phase coherence during speech, specific to the theta band (P<0.05, timepoint-by-timepoint mixed effects analysis relative to the pre-speech baseline, N=1214 electrode-pairs across 14 subjects, FDR-corrected; Fig. 2G). The spatial extent of this theta-band phase coherence was broad, encompassing multiple sites within the speech production network, with strongest coherence localized to the bilateral vSMC and extending into prefrontal, supramarginal, and superior temporal regions (Fig. 2H).

Notably, this mesoscopic, speech-related increase in phase coherence was significantly stronger during production than during passive listening—arguing against an acoustic origin. Moreover, comparable coherence magnitude was observed during spontaneous storytelling, indicating that the effect generalizes to natural, self-generated speech beyond reading (Fig. S5D-E).

While coherence quantifies the strength of coupling, it does not distinguish whether sites oscillate in synchrony or with systematic phase offsets. To disambiguate whether the observed coherence reflected true synchrony (zero phase) or consistent phase offsets across sites, we quantified the mean phase difference of each speech-responsive electrode during articulation relative to the most ventral SMC site in each subject. Across all speech-responsive sites, we observed a bimodal distribution of phase differences: the majority exhibited near-zero phase coupling (0° ± 45°; 827/1328 electrodes, 62%), while a substantial subset showed antiphase coupling (180° ± 45°; 277 electrodes, 21%) (Fig. S5). The functional significance of these phase relationships is explored in the following sections.

### Theta/High-Gamma phase amplitude coupling during speech

To understand how coherent theta oscillations might shape the timing of neuronal activity across the premotor speech network, we next asked whether the local theta phase modulates the magnitude of HG activity—an established marker of local population spiking activity (53–55)— in speech-responsive sites. Given the broad increase in theta phase coherence described above, even subtle theta-coupled enhancement of HG amplitude, when aggregated across coherently oscillating premotor sites, could give rise to a prominent pulsatile output signal. Much like crickets synchronizing their weak calls into a unified, pronounced pulsatile sound that can be heard from a distance, this coordination may serve to temporally align, integrate, and amplify neuronal signaling, focusing distributed premotor spiking within a theta-structured excitability window. In the context of articulatory control, such convergence is likely essential for broadcasting a unified, synergistic motor command to multiple articulators simultaneously.

To test this idea, we computed the phase-amplitude coupling (PAC) across speech-responsive sites using Tort’s Modulation Index (MI), as proposed by (56, 57) (see Methods). We computed this index for each speech-responsive site individually and assessed statistical significance by comparing it to a surrogate distribution generated from 5,000 random circular shifts of the theta phase time series (Fig. S6; see Methods).

A widespread and statistically robust PAC was observed, with HG amplitude significantly modulated by local theta phase in 76.2% (975/1,280) of speech-responsive electrodes (P < 0.05, FDR-corrected; see Fig. S6 for details). Across electrodes, HG amplitude was highest near the theta trough (Fig. S6). It should be noted that this theta-rhythm modulation was often superimposed on slower, seconds-long HG activation profiles time-locked to sentence onset (see Fig. S1 for examples).

### Quasi-rhythmic modulation of vocal tract kinematics during continuous speech

The large-scale theta phase coherence during speech, coupled with robust theta–HG PAC, suggests that a significant portion of speech-related premotor population activity is synchronized by the theta rhythm. At the mesoscopic scale, such in-phase oscillatory modulation can rapidly aggregate into a pulsatile neuronal signal at the output stations of the circuit, where motor commands are integrated and transmitted to the articulators. A direct prediction that follows is that the kinematics of vocal tract movements, encoded by the vSMC(1, 5, 6), should exhibit pulsatile dynamics coupled to the sensorimotor rhythm.

**Fig. 3.**
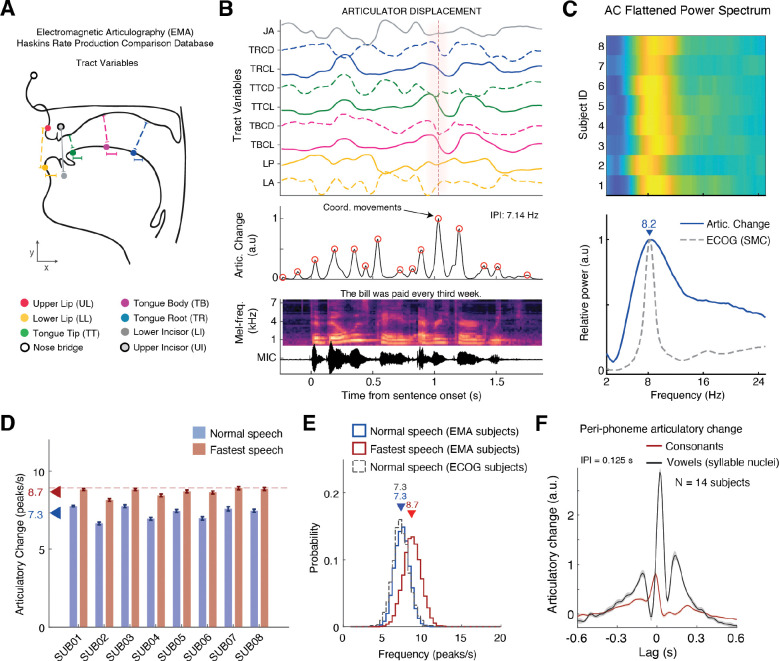
Quasi-rhythmic, pulse-like modulation of articulator velocity during continuous speech. (A) Example of articulatory kinematics for the jaw, lips and tongue monitored by Electromagnetic Midsagittal Articulography (EMA) during continuous speech production. (B) Top: Kinematic trajectories of the measured tract-variables including lip aperture and protrusion, tongue root, body and tip positions, and jaw angle (see Methods). Middle: We found semi-rhythmic temporal modulation of articulatory movements characterized by abrupt, pulse-like, changes in the sum of squared velocities across articulators. These brief pulses reflect coordinated movements associated with phonemes production (58, 59). Bottom: Mel-spectrogram of produced acoustics. **(C)** Mean power spectrum showing a clear concentration of power around ~8 Hz. The gray trace depicts the average power spectrum in SMC during articulation, derived from the ECoG subjects for comparison. **(D)** Analysis of the inter-peak intervals (IPI) of the AC time series during normal speech (~4 syllables/s; blue bars) reveals a highly consistent median AC pulse rate across individuals (mean ± SE: 7.30 ± 0.15 peaks/s). When participants were instructed to speak as rapidly as possible without errors (red bars), the median AC pulse rate increased but remained centered below ~9 Hz (8.66 ± 0.09 peaks/s). Error bars represent ± SEM across sentences. **(E)** Comparing the distribution of sentence-level AC pulse rates during normal speech in ECoG participants—where articulatory kinematics were estimated using AAI (dashed gray)—to those obtained from actual EMA measurements (blue) revealed no significant difference (P = 0.357, Wilcoxon rank-sum test; ECoG group: Med = 7.3, IQR = 1.4 pulses/s, N = 14; EMA group: Med = 7.3, IQR = 1.5 pulses/s, N = 8). For reference, the red distribution shows the upper bound of AC pulse rates, derived from EMA participants instructed to speak as quickly as possible. While this distribution shifts rightward, it remains centered within the theta frequency range (mean±SE: 8.66 ± 0.09 pulses/s). **(F)** Fitting a linear model to predict AC from produced phonemes (see Methods) revealed that the production of individual consonants (red) is associated with a single AC pulse. Phonemes forming the nucleus of a syllable (primarily vowels) elicit a larger AC pulse surrounded by secondary pulses, reflecting the preceding (onset) and following (coda) consonants that together with the nucleus form a syllable. Notably, the inter-peak interval of 0.125 s gives rise to a brief oscillatory pattern at 8 Hz.

To test this prediction, we first analyzed electromagnetic midsagittal articulography (EMA) data from the Haskins Production Rate Comparison database (see Methods). These EMA recordings capture the kinematics of the upper vocal tract articulators (i.e., jaw, lips and tongue), with high temporal resolution (~100 Hz), during the production of English sentences similar to those used in our study (Fig. 3A-B). Consistent with previous reports on rhythmic modulation of articulatory velocity during continuous speech (58–60), we found that the overall Articulatory Change (AC) during speech, namely, the sum of squared velocities (i.e., total speed) across the measured tract-variables, demonstrate rhythmic “pulses”, during which several articulators move together in synergy to form a constriction gesture (5, 58) (Fig. 3B).

In alignment with our prediction, the rate of these pulses during fluent speech consistently centered around 8 Hz. This was evident in both the flattened power spectrum of the AC time series (Fig. 3C) and the distribution of inter-peak intervals (Fig. 3D; see Methods). This quasi-rhythmic pulsatile behavior closely aligns with the mean peak frequency of the sensorimotor cortical rhythm across subjects (8.2 ± 0.7 Hz; Fig. 3C). Notably, when the EMA subjects were instructed to produce the same sentences as quickly as possible without errors, the rate of AC pulses increased but remained centered consistently below 9 Hz, i.e., within the sensorimotor theta range (median pulse rate averaged across subjects ± SE: normal speech [4 syl/s], 7.30 ± 0.15 pulses/s; fastest speech [5.8 syl/s], 8.66 ± 0.09 pulses/s; Fig. 3D).

While the EMA analysis revealed that speech movements exhibit rhythmic pulses in the theta range, these data alone cannot establish a direct relationship between cortical oscillations and vocal tract kinematics, as the neural and articulatory signals were recorded from different individuals. To determine whether AC pulses are temporally coupled to the ongoing sensorimotor theta rhythm, both signals—neural and kinematic—must be measured within the same subject. However, direct measurement of articulatory movements using EMA is impractical in ECoG subjects due to clinical and methodological constraints associated with bedside recordings. To overcome this limitation, we employed a deep learning-based acoustic-to-articulatory inversion (AAI) method (5, 61), which infers the trajectories of the jaw, lips, and tongue directly from speech acoustics with high temporal precision. Fig. S7 shows example kinematic trajectories from a representative ECoG subject, along with the mean AC pulse rates across individual subjects.

To validate the fidelity of AAI-derived kinematics, we compared the AC pulse rates in ECoG subjects to those obtained from the EMA dataset during normal speech. The results showed no significant difference in median AC pulse rate between the two groups (ECoG vs. EMA: P = 0.36, Wilcoxon rank-sum test; Fig. 3E), confirming that the AAI approach preserves the fine-grained rhythmic structure of articulatory behavior during continuous speech.

### Articulatory Change in Relation to Phoneme and Syllable Production

To better understand the nature of the observed articulatory pulses, we next sought to characterize how the AC signal aligns with the elementary units of speech—phonemes and syllables. While prior work has characterized AC in relation to various articulatory landmarks (58), we extend this by isolating the fine-grained temporal profile of AC during the production of individual consonants versus vowels (i.e., syllable nuclei). Since the syllable is considered a fundamental unit of speech timing (60, 62–64), and phoneme transitions are densely concentrated around its nucleus (58), we hypothesized that AC would exhibit a temporally structured sequence of pulses clustered around the vowel. As the most sonorous segment and the point of maximal vocal tract opening, the syllabic nucleus provides a natural temporal anchor for adjacent consonantal gestures (65), particularly during rapid consonant–vowel (CV) and vowel–consonant (VC) transitions. We therefore expected a prominent AC pulse aligned with the vowel, flanked by smaller pulses corresponding to the surrounding consonants.

To test this, we fit a linear multivariate temporal response function (mTRF) encoding model (66) to predict AC magnitude from phoneme onsets (see Methods). This approach allowed us to isolate the AC profile associated with individual consonantal gestures and compare it to the pattern emerging around the vowel.

Consistent with the idea that the syllabic nucleus acts as a temporal anchor, the model revealed a structured, quasi-rhythmic cluster of articulatory pulses centered around the vowel during syllable production. Fig. 3F plots the predicted AC magnitude in a −0.6 to +0.6 s window aligned to the onset of consonants (red) and vowels (black). Whereas individual consonants were tied to a single pulse, vowels were associated with a triplet of pulses corresponding to the syllable’s onset, nucleus, and coda. Notably, the mean inter-pulse interval in this triplet was 0.125 s—closely matching the 8.2 Hz sensorimotor theta rhythm. Supporting this, we also observed a strong correlation between AC pulse rates and syllable rates across subjects (R^2^ = 0.94, P < 10^−7^; Fig. S7D), reinforcing the notion that AC pulses directly reflect the execution of articulatory gestures.

### Vocal-tract movements coupled to sensorimotor theta phase

If sensorimotor theta oscillations coordinate the timing of vocal tract movements during speech, then AC pulses should exhibit significant coupling to specific phases of the theta cycle. Since HG activity across the speech production network is strongly modulated by theta—peaking in unison around the trough (Fig. S6)—motor output is expected to reach local maxima shortly after these mesoscopic excitability windows. Accordingly, we predict that AC magnitude will transiently peak following these coordinated cortical activations.

To test this prediction, we binned the instantaneous theta phase into 24 bins (0 to 2π, 15° wide) and computed the average AC within each phase bin. If articulatory movements occur independently of theta phase, AC should be uniformly distributed across bins. To assess the coupling strength between theta phase and articulatory movements and determine statistical significance, we used Tort’s Modulation Index (56, 57) and a non-parametric shuffling test, following the same approach used for the PAC analysis above (see Methods).

**Fig. 4.**
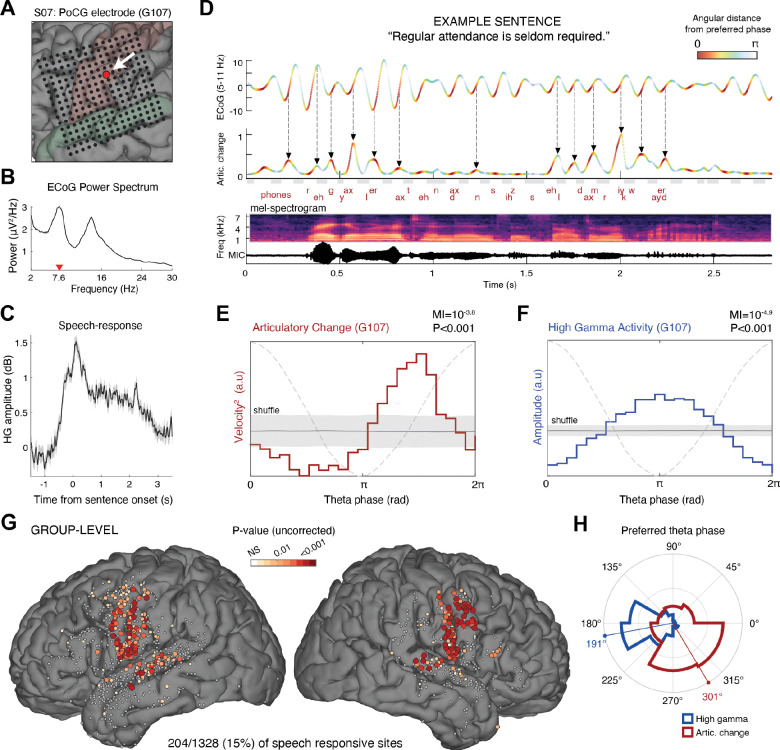
Articulatory change during continuous speech is coupled to the sensorimotor theta cycle. **(A)** Electrode’s anatomical location, **(B)** Power spectrum of the raw ECoG signal. Notice the spectral peak at 7.6 Hz (red triangle). **(C)** Mean HG response relative to sentence onset. **(D)** Example of a sensorimotor theta oscillation (top; 5–11 Hz bandpass filtered) and concurrent articulatory change (AC; middle) during the articulation of a representative sentence (produced phonemes, speech amplitude, and a mel-spectrogram are presented at the bottom). The theta rhythm was measured at a representative postcentral speech-responsive site described in panel a-c. The two timeseries are color-coded using the same colormap, indicating the angular difference from the preferred theta phase at this site, i.e., the phase at which articulatory change was maximal on average (see panel E). **(E-F)** We used Tort’s Modulation Index method (56) along with a shuffling procedure (see Methods) to statistically assess the coupling between theta phase and AC (E, red trace), and between theta phase and HG amplitude (F, blue trace). In this representative electrode, AC was significantly coupled to theta phase (MI=10^−3.8^, P<0.001), peaking a quarter cycle after the trough, while HG amplitude was maximal at the trough. Gray shaded area represent mean ±SD of the shuffled data; the dashed line represents the theta cycle. **(G)** Spatial distribution of recording sites where AC was significantly coupled to the theta rhythm (P<0.05, FDR corrected, 204/1328 (15%) of the speech responsive electrodes across 14 subjects). Note the anatomical specificity of the effect, clustering within SMC and the posterior STG. Non-significant electrodes are shown in light gray. **(H)** Polar histogram showing theta phase preferences for AC (red; P<10^−10^, Rayleigh test) and HG activity (blue; P<10^−44^, Rayleigh test). Only significant AC-theta coupled electrodes were included (N=204 sites). Notably, a consistent phase difference is observed between HG activity and A.C, with articulatory movements consistently preceded by HG activation within the theta cycle.

Fig. 4A-F presents the MI analysis for an example vSMC recording site, showing significant and consistent increases in AC coupled to the late phase of the theta cycle. Fig. 4D displays a single trial example from this site, demonstrating individual AC pulses that emerge consistently toward the end of concurrent theta cycles. Across 1,328 cortical speech-responsive electrodes in 14 subjects, we identified 204 sensorimotor sites (15%) with significant theta-AC coupling (P<0.05, FDR corrected; Fig. 4G). These electrodes formed a tight cluster along the central sulcus—spanning the pre- and post-central gyri and extending into posterior STG.

Notably, while the theta rhythm was observable and coherent across multiple sites within the speech production network, only this sensorimotor subset consistently translated the cyclic excitability windows into phase-locked motor output, highlighting its more direct role in articulatory execution.

In most sites (69%), the preferred phase—where AC was maximal—concentrated at the second half of the theta cycle (mean ± s.d.: 301°± 84°, P<10^−10^ Rayleigh’s test, N=204 electrodes; Fig. 4H and Fig. S8), approximately a quarter cycle after the HG amplitude enhancement found around the trough (preferred phase angular difference ± s.e., AC-versus-HG: 93° ± 5.8°, mean resultant length (MRL)=0.49, P<10^−15^ Rayleigh’s test; Fig. S8).

Interestingly, sites where AC was coupled to an earlier theta phase—specifically, before the trough—were consistently clustered in the dorsal portion of the ventral sensorimotor cortex (vSMC) (Fig. S8). This region has been implicated in articulatory motor planning (67), speech inhibition (68), and laryngeal control (7). These dorsal sites exhibited a theta rhythm that was nearly in anti-phase relative to more ventral “late-coupling” sites (mean phase ± SE relative to vSMC reference site: “early-coupling” electrodes (N = 26): −173° ± 24.5°; “late-coupling” electrodes (N = 90): 7° ± 4.6°; early vs. late: P < 0.001, Watson’s U^2^ test; see Fig. S8 for details).

This bimodal distribution of theta phases is expected under the assumption that neighboring sites in the sensorimotor cortex are interconnected through both excitatory and inhibitory pathways (69). The existence of two anti-phase oscillators aligns closely with the coupled oscillator planning model (65)—a prominent model of speech timing positing that two theoretical oscillators, offset by 180°, govern the relative timing of articulatory gestures during syllable production. Within this framework, the in-phase/anti-phase theta pattern appears well suited to support both (i) transitions between the syllabic nucleus and coda elements (65), and (ii) the sequencing of complex consonantal gestures that require precisely timed transitions from full closure to release (3, 62), as in oral plosives. Producing two such consonants in succession may leverage the 180° phase offset to schedule the second gesture half a cycle after the first, helping to prevent overlap—particularly when the same articulator is involved. Future research is needed to further elucidate the functional significance of this biphasic pattern across diverse articulatory contexts.

### Theta cycles orchestrate sequential premotor activations during articulation

The observation of systematic leading and lagging theta phases across sensorimotor sites suggests a sequential dorsoventral sweep of activation within each theta cycle. Specifically, early-phase sites would show theta-coupled HG activation first, followed by late-phase sites as the oscillatory cycle progresses. These theta-orchestrated activity sweeps naturally invite comparison to the phenomenon of “theta sequences” described in the rodent hippocampus, where sequential neural activity that unfolds over hundreds of milliseconds during behavior—e.g., as an animal moves through space—emerges in the same order but on a compressed timescale (~125 ms) during individual theta cycles (70).

In these rodent studies, the greater the distance between two neurons’ place-field centers, the larger the difference in their preferred firing phases, indicating that spatial trajectories are mapped onto theta phase (70, 71). Motivated by this analogy, we asked whether a similar relationship between behavioral and theta-compressed timescales exists for sensorimotor sequences encoding articulatory movements. Specifically, do electrodes that activate earlier relative to the onset of articulatory gestures also exhibit earlier relative theta phases?

To address this, we fitted a linear mTRF encoding model that predicts HG amplitude from articulatory kinematics (see methods). Crucially, this modeling approach enabled us to isolate the HG response associated with AC modulation while controlling for other kinematic features. It also allowed us to directly compare the peri-AC activation profile across electrodes with leading versus lagging theta phases. Importantly, since the encoding model operates exclusively in the time domain, it is entirely ‘blind’ to the relative theta phase differences across sites. By comparing these time-domain peri-AC activation profiles with the independent phase relationships described earlier, we can assess whether phase order aligns with temporal order—that is, whether early-phase sites precede late-phase sites not only within the ~125 ms window of a theta cycle, but also across the continuous, hundreds-of-milliseconds timescale of articulatory execution—resembling the theta sequences observed in rodents.

**Fig. 5.**
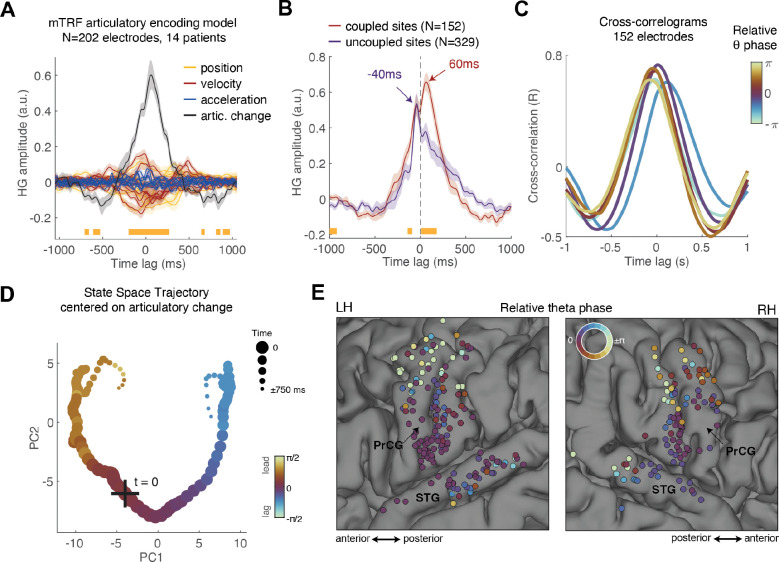
Sequential premotor cortical activations organized by theta phase. **(A)** HG activation profiles obtained from the mTRF articulatory encoding model, fitted to electrodes exhibiting significant theta-movement coupling (P < 0.05, FDR-corrected; N = 202 electrodes across 13 subjects). The weights corresponding to the Articulatory Change (AC) feature were significantly greater than those of any single-articulator feature (P < 0.05, FDR-corrected; timepoint-by-timepoint mixed-effects analysis comparing AC against all other features). Shaded error bars represent SE estimated from the mixed-effects model. **(B)** Peri-AC activation profiles averaged across ‘theta-coupled’ and ‘theta-uncoupled’ electrodes with significant unique variance explained by the AC feature. HG amplitude was significantly higher in ‘theta-coupled’ electrodes during the execution of articulatory movement (one-sided timepoint-by-timepoint mixed-effects analysis, P<0.05, FDR corrected). Shaded error bars represent SE estimated from the mixed-effects model. **(C)** Cross-correlograms between the peri-AC HG activation profile of each electrode and the average profile across all electrodes, computed separately for six relative-phase bins spanning – π to π. The cross-correlogram peaks shift systematically with the electrodes’ relative phase. **(D)** State-space trajectory computed by projecting the peri-AC activation patterns onto the first two principal components. The time points along the trajectory are color-coded based on the relative theta phase of the top 10% of electrodes with the strongest PC1 and PC2 weights at each time point. **(E)** Anatomical map of theta phase relative to the most ventral SMC site, including 283 electrodes in the SMC and STG exhibiting significant (P<0.05, uncorrected) theta-movement coupling across 14 subjects.

Across the electrodes that exhibited significant theta–movement coupling (P < 0.05, FDR corrected; N = 202 electrodes), we found a robust HG response time-locked to the onset of AC increase (Fig. 5A and Fig. S9). This peri-AC cortical activity was significantly stronger than the activation driven by any other kinematic feature, as shown by a timepoint-by-timepoint mixed-effects analysis (AC vs. all other features: P < 0.05, FDR corrected; Fig. 5A). These findings are consistent with our previous work demonstrating preferential encoding of both articulator speed (6) and multi-articulator gestures over single-articulator movements in the vSMC (5). Additionally, AC explained a significant proportion of unique variance across subjects (1 ± 0.09%, P < 0.0004, Wilcoxon signed-rank test; N = 14), confirming that it constitutes a key kinematic feature robustly encoded in the SMC.

Next, we investigated whether electrodes exhibiting theta-movement coupling also demonstrated stronger AC encoding compared to those without such coupling. We pooled electrodes with significant unique variance explained by AC (P < 0.05, FDR corrected) into two groups: ‘theta-coupled’ electrodes (MI significance of P < 0.05; N = 157 electrodes across 14 subjects) and ‘theta-uncoupled’ electrodes (P > 0.1; N = 329 electrodes across 14 subjects). Comparing peri-AC activation profiles revealed that theta-coupled electrodes were more strongly driven by AC (P < 0.05, FDR corrected; timepoint-by-timepoint mixed-effects analysis of beta weights; Fig. 5B). In the ‘theta-coupled’ group, HG activity began increasing 300–400 ms before the AC and peaked 60 ms afterward, aligning with both premotor and execution-related activation. Conversely, in the ‘theta-uncoupled’ group, activity primarily anticipated the AC increase but culminated 40 ms before it, suggesting a predominant role in premotor preparation rather than the control of ongoing movement.

To test whether the relative theta-phase in ‘theta-coupled’ electrodes correlates with the onset of peri-AC response, we computed a cross-correlogram between each electrode’s peri-AC response and the grand-average response profile across all electrodes. We took the peak of this cross-correlogram as the electrode’s relative latency. We then assessed the correlation between these latencies and the electrodes’ theta phases, measured relative to each patient’s most ventral SMC site (see Methods).

This analysis revealed a robust circular–linear correlation between theta phase and the latency of peri-AC activation across the SMC (ρ = 0.41, P < 10^−5^; N = 152 electrodes), implicating theta phase in the temporal organization of vocal-tract control at the mesoscopic (multi-electrode, circuit-level-) scale. To visualize this relationship, we grouped electrodes into six phase bins spanning −π to π and averaged their cross-correlograms. As shown in Fig. 5C, the correlogram peak shifts progressively across phase-bins: electrodes with leading theta phase show earlier peri-AC activation. This relationship is further illustrated in Fig. 5D, which shows the state space trajectory of peri-AC activation in the SMC, with each point color-coded by the mean theta phase of the 10% most active electrodes (see Methods). Finally, Fig. 5E maps relative theta phase across ‘theta-coupled’ electrodes in SMC and STG, revealing a clear phase gradient along the SMC’s dorsoventral axis.

Lastly, to further quantify the relationship between the theta cycle and the continuous “behavioral” time, we computed the correlation between the latency differences across electrode pairs—estimated from cross-correlogram peaks—and their corresponding theta phase differences. Consistent with the preceding analyses, we found a highly significant correlation (*r* = 0.22, *P* < 10^−16^; Fig. S9), confirming that relative timing of peri-AC activation mirrors the phase structure of the ongoing theta rhythm.

### Consecutive theta cycles orchestrate successive movements during syllable production

**Fig. 6.**
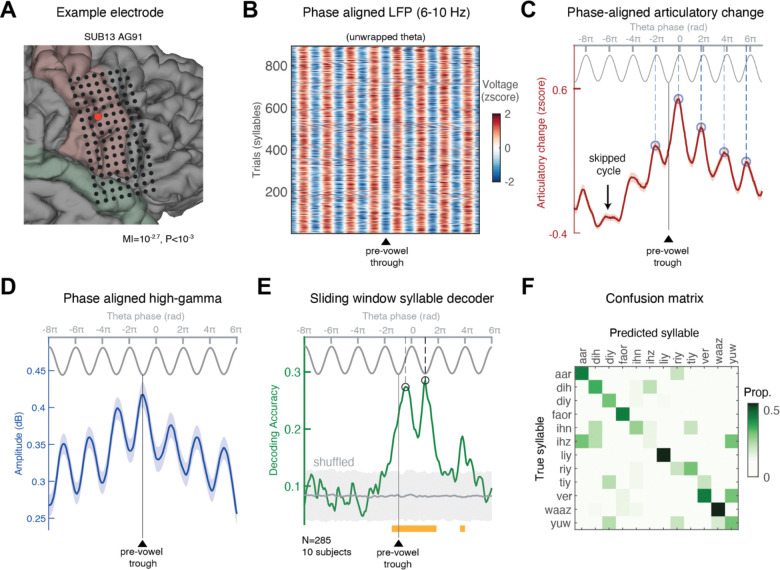
Consecutive theta cycles time articulatory movements during syllable production. **(A)** Anatomical location of a representative SMC site showing significant theta-movement coupling. **(B)** Each trial was aligned to the theta trough preceding the vowel (indicated by a black triangle). The theta phase was unwrapped and interpolated along a common phase axis spanning −9π to 7π (see Methods). Only syllables separated from the preceding one by at least 250 ms were included, allowing examination of whether theta–AC coupling persisted across brief pauses involving skipped cycles. **(C)** Articulatory change (AC) was interpolated along a uniform phase axis using the unwrapped instantaneous theta phase, as described in (B). The phase-aligned AC was then averaged across syllables. The analysis revealed consecutive AC pulses tightly locked to a specific phase of the theta cycle. In this electrode, AC peaked slightly before the theta peak (dashed blue vertical line). The arrow marks a cycle approximately 250 ms before syllable onset during which no articulatory movement occurred. Nevertheless, the subsequent movement remained coupled to the theta rhythm, consistently emerging at the preferred phase. **(D-F)** A sliding-window SVM syllable decoder was trained on the 12 most common syllables in the dataset, using 285 electrodes with significant theta-movement coupling, pooled from 10 subjects. (D) Phase-aligned HG amplitude, processed as described in (B), displayed pulsatile dynamics tightly coupled to theta troughs. (E) Decoding accuracy peaked during the two cycles overlapping with the produced syllable, slightly after the theta trough (peak phase: 4.07±0.15 rad). The gray-shaded area represents shuffled data (mean ± 95% CI), significant bins highlighted in orange. (F) Confusion matrix, computed by combining classifier outputs across the significant phase bins.

Syllable production involves rapid transitions between successive constriction gestures associated with individual phonemes. Behaviorally, the temporal profile of AC relative to syllable nucleus suggests quasi-rhythmic transitions between gestures (Fig. 3F). Does the sensorimotor theta oscillation coordinate these transitions? To address this question directly, we epoched the ECOG and AC timeseries relative to syllable nucleus (i.e., vowels). In this analysis, we only included syllables that were well isolated in time, i.e., followed a period of at least 250 ms with no significant articulatory movement (see Methods). This resulted in a total of several hundred syllables per subject. For simplicity, the analysis was performed on a single sensorimotor site in each subject—the one that exhibited the strongest coupling between theta and AC (Fig. 6A-C).

We then aligned each epoch to the theta trough closest to the syllable nucleus and extracted the unwrapped analytic theta phase for each epoch (see Methods). To ensure consistent phase alignment from beginning to end, we resampled the continuous phase series through linear interpolation onto a uniform phase axis ranging from [−9π to 7π]. The same interpolation was applied to the other signals, allowing us to average both the local population spiking activity (HG) and the concurrent AC across syllables while preserving their fine-grained alignment with the theta cycle (thus avoiding phase cancellation).

We found that the AC pulses associated with syllable production exhibited clear pulsatile dynamics that were tightly aligned with the ongoing theta phase, extending across several consecutive cycles. This is shown in Fig. 6C, which depicts the average AC in a representative site (see Fig. S10A-B for an additional example). Notably, because the analyzed syllables were preceded by brief articulatory pauses (see Methods), we were able to assess whether the first articulatory movement after such pauses “waited” for the next theta cycle. As shown in Fig. 6C, even after skipping an entire theta cycle, the subsequent movement consistently occurred at the preferred theta phase of that electrode.

For the group-level analysis, we centered the epochs on the preferred phase of each electrode (as determined by the MI analysis in Fig. 4) and averaged across subjects (Fig. S10C). For statistical testing, we generated surrogate data by circularly shifting the theta phases in each trial by a random amount 50 times (see Methods). Comparing the empirical and surrogate data revealed significant AC pulses emerging at the preferred phase across consecutive theta cycles—both in the cycle immediately preceding the syllable nucleus and in the cycles that precede or follow it. To confirm that this effect arises from the coupling of individual AC pulses, and not from a subset of particularly prominent movements, we also computed the group-level phase histogram (i.e., probability distribution) of AC peaks, which captures only the timing of the pulses rather than their amplitude. The results of this analysis, shown in Fig. S10E, indicate significant coupling of AC pulses to theta phase, irrespective of amplitude.

### Decoding syllabic content along the theta cycle

So far, we have shown strong coupling between theta phase, neuronal activity, and vocal tract movements. However, a key open question is whether the mesoscopic sensorimotor HG activity organized by the theta cycle actually represents speech content. Put differently, would a classifier trained to decode syllable identity from vSMC population activity exhibit performance that systematically varies with theta phase?

For the analysis we selected the 12 most common syllables in our dataset, i.e., those with a sufficient number of repetitions (N=10) to train a classifier (e.g., /ri/, /ɪz/, /fɔr/, /lɪ/, /ju/, etc.). We applied the phase-alignment procedure outlined above and centered all data epochs to the theta trough near the nucleus of the syllables. We then pooled together all electrodes that exhibited robust coupling (P<0.05, uncorrected) between theta and vocal-tract movement (n=285 across 10 subjects) and constructed a group-level matrix of HG activity during syllable production (electrodes × phase × trials). Crucially, like in the analysis above, this matrix aligned the activity in individual trials to a common phase axis ranging from [−9π to 7π], allowing us to link decoding performance to the instantaneous theta phase rather than time. Fig. 6D depicts the average HG amplitude across trials and electrodes, showing a strong pulsatile signal peaking at the trough of each theta cycle—as expected from the PAC described in Fig. S6.

Next, we trained a sliding-window Support Vector Machine (SVM) classifier (see Methods), with a 0.3-radian window, to decode syllable identity from HG activity patterns.

Fig. 6E–F presents the results of this analysis. Decoding performance was significantly above chance specifically during theta cycles that overlapped with syllable production (actual vs. shuffled labels: *P* < 0.05, FDR-corrected). Accuracy peaked shortly after the theta trough—i.e., when neuronal excitability is highest (Fig. 6D; phase of peak accuracy: 4.07±0.15 radians, ~53° after the trough). When classifier outputs were combined across the significant phase bins (weighted by their mean accuracy), decoding accuracy reached 35.8% (chance = 8.33%). Fig. 6F displays the confusion matrix integrated across these significant bins. These findings underscore the role of theta oscillations in orchestrating mesoscopic, multi-site, sensorimotor activity that collectively represents the executed articulatory gesture during speech.

### Reduced Theta-Movement Coupling linked to Speech Errors

Our results identified strong coupling between theta and movement during fluent speech. If this coupling is important for orchestrating the complex motor sequences underlying speech, then we would expect it to be disrupted on trials where speech contained articulatory errors.

To test this, we analyzed utterances containing articulatory errors or speech repairs, focusing on electrodes showing significant theta–movement coupling. Subjects with fewer than 10 error trials were excluded. For each remaining participant, a control set of fluent trials was randomly sampled to match the error trials in syllable rate, duration, and sample size. This sampling was repeated 200 times to ensure stability, and the median of these resamples was used for analysis (Fig. S11).

Theta power did not significantly differ between fluent and error trials (*P* = 0.16, mixed-effects analysis; 117 sites, 7 subjects; Fig. S11A), suggesting that speech disruptions were not driven by changes in oscillatory power. Instead, it was the strength of theta–movement coupling (theta/AC MI) that was significantly reduced during speech errors (*P* = 0.001, mixed-effects analysis; Fig. S11B). Moreover, while the preferred theta phase was highly consistent across odd and even samples of fluent trials, error/repair trials exhibited greater variability in phase preference across electrodes. This was confirmed by significantly greater phase consistency across electrodes between two independent sets of fluent trials, compared to the consistency observed between fluent and error trials (resultant vector length: fluent = 0.99, errors/repairs = 0.73; P < 10^−6^, Fisher dispersion test; Fig. S11C-D). These findings suggest that speech errors are accompanied by a momentary disruption in the coupling between theta phase and articulatory movement, consistent with the idea that stable theta–movement coupling supports fluent speech.

### Theta Periodicity Predicts Individual Differences in Speech Rate

Lastly, the notion of sensorimotor theta as an internal timing mechanism for speech prompts a key question: are individual differences in speech rate driven by faster oscillations, or by more precise rhythmic timing? In other words, do fast speakers have a faster internal sensorimotor rhythm, or does their “metronome” tick more reliably, enabling more effective and precise coordination?

To address this, we analyzed the autocorrelation function (ACF) of the theta-band signal from the top five electrodes in each subject that showed robust theta–movement coupling. We first tested whether individual speech rate was associated with the frequency of the sensorimotor theta rhythm, but found no significant correlation (r = 0.18, *P* = 0.54, n.s.). However, when we assessed the periodicity of the oscillation—defined as the magnitude of the secondary ACF peak—we observed a striking relationship (Fig. S12A).

Subjects were divided into fast and slow speakers using a median split based on their mean syllable rate during the task. Fast speakers exhibited significantly stronger theta rhythmicity (*P* = 0.0006, rank-sum test; Fig. S12B), as indicated by more pronounced secondary ACF peaks (Fig. S12A–B), and greater theta-band power (P = 0.0012, rank-sum test). These results suggest that faster speech is not driven by a higher theta frequency, but by a more pronounced and temporally stable underlying rhythm.

Furthermore, theta rhythmicity correlated positively with speech rate during both sentence reading (Spearman’s ρ=0.79, P=0.001) and natural conversation (Spearman’s ρ=0.66, P=0.013; Fig. S12C–D). For the latter, we computed median articulation rates from spontaneous conversational utterances (mean length: 11 ± 3.1 words), excluding speech pauses. Together, these findings support the hypothesis that individual differences in speech rate are not driven by faster sensorimotor oscillations, but rather by greater stability and periodicity of the oscillations—consistent with the view of theta rhythm as an intrinsic timing mechanism that varies in strength and reliability across individuals.

## Discussion

In this study, we leveraged high-density electrocorticographic recordings in epilepsy patients to investigate the role of intrinsic neural oscillations in speech motor control. We identified a prominent theta rhythm (6–10 Hz) within the sensorimotor speech production network that supports the motor coordination required for fluent speech. Analysis of jaw, lip, and tongue movements during articulation revealed quasi-rhythmic, pulse-like modulations of articulator velocity, occurring ~7–8 times per second on average. These motor pulses, reflecting rapid vocal-tract gestures, were tightly locked to specific phases of the cortical theta rhythm—with consecutive theta cycles timing successive phoneme-level gestures during syllable production. At the mesoscopic scale, the theta cycle orchestrated multi-site sensorimotor HG activation patterns, with syllabic identity optimally decodable shortly after theta troughs.

Our results thus demonstrate that mesoscopic representations of articulatory gestures are organized into precisely timed “pulses” of motor commands orchestrated by the theta cycle. Momentary disruptions in the coupling between theta-phase and articulatory movements predicted speech disfluencies, and individuals with stronger theta rhythmicity exhibited significantly faster speech rates.

These findings reveal a previously unrecognized role for theta oscillations in speech motor control, serving as a shared timing signal—an intrinsic temporal scaffold—that enables precisely timed, distributed control across multiple articulators moving in synergy. Such precise timing is essential for scheduling rapid transitions between articulatory gestures during fluent speech.

More broadly, our results highlight the critical role of intrinsic oscillations in achieving millisecond-scale coordination of distributed neuronal activity across mesoscopic brain circuits, providing direct empirical support for several existing theories on the function of neural oscillations (12, 16, 36, 39, 43, 72–78).

Our findings also shed light on dynamics of phase coherence and PAC in cortical circuits (79). The co-occurrence of these phenomena suggests that, at the output stations of the oscillating circuit, spatially distributed activity may converge into a strong, distinct pulsatile signal. These mechanisms channel spatially distributed neuronal activity into discrete excitability windows aligned to specific oscillatory phases—a process particularly advantageous for coordinating muscle synergies with precise timing (76). Our demonstration of this mechanism in articulatory control parallels previous findings in the control of human hand and finger movements, where quasi-rhythmic kinematic pulses at ~8 Hz have also been reported (80). Similarly, recent work in bats shows that wingbeat movements are highly rhythmic at ~8 Hz (81). Together, these parallels suggest that the underlying premotor oscillatory mechanism may represent a general organizing principle for dexterous motor control, potentially extending beyond speech to other finely timed behaviors involving the hands, limbs, and orofacial effectors, and generalizing across species.

Prior work, dating back to early scalp and intracranial EEG studies (82–84), identified a rhythmic signal in the 8–13 Hz range, commonly referred to as the Rolandic mu rhythm or central alpha. This cortical rhythm has been traditionally studied in the context of motor tasks involving simple, discrete movements of the hands, feet, or tongue (16, 50, 85–89). In these early studies, sensorimotor oscillations often exhibited a time-locked power attenuation at movement onset, leading to their common characterization as the ‘idle rhythm’ of the sensorimotor cortex—most prominent in the absence of voluntary movement (16, 90). Later studies revealed more heterogeneous patterns, including both amplitude increases and decreases as well as inter-regional phase synchronization akin to what we observed—suggesting a more complex functional role with substantial task-dependent variability (36, 49, 90, 91). Our findings extend this foundational work. By analyzing sensorimotor oscillations intracranially under the rapid and sustained motor demands of fluent speech production, we uncover a previously unrecognized role for these intrinsic sensorimotor rhythms in motor coordination. To distinguish this signal from the canonical mu rhythm—and to reflect its slightly lower frequency range (6–10 Hz) while maintaining alignment with related findings in animal models (17, 22, 30)—we referred to it as sensorimotor theta.

Our results reinforce the emerging view that theta rhythms are central to coordinating self-initiated movement and integrating it with cognitive processing—both in rodents (17, 20, 22, 24, 30, 92, 93) and non-human primates (19, 21), underscoring the generalizability of these oscillatory dynamics across species and motor systems.

In line with this broader integration, we also find evidence of sensorimotor theta oscillations resonating within hippocampal circuits (Fig. 1E), suggesting a potential role in coordinating cortico-hippocampal communication during speech—a topic for future investigation. Relatedly, recent work (94) reported an increase in the coupling between neuronal spiking in the subthalamic nucleus and cortical theta rhythms during syllable production, supporting the idea that widespread theta phase coherence facilitates cortical–subcortical interactions during speech.

Critically, the intrinsic sensorimotor theta rhythm we report emerges independently of acoustic input. It is evident across diverse behavioral states, including silent rest, and maintains a stable central frequency despite large variations in speech syllable rate. A silent-miming control further dissociates this rhythm from acoustic feedback, underscoring a self-sustained premotor origin.

This finding provides a neurophysiological perspective that complements substantial EEG and MEG research implicating theta-band oscillations in auditory cortex during speech perception (60, 95). In that work, theta activity within auditory cortex resets its phase to speech onsets and tracks the temporal envelope of the acoustic signal—processes linked to speech intelligibility (96–98). Perturbing the rhythmic structure of speech in the theta range likewise impairs intelligibility (99, 100). Although the relative contributions of endogenous entrainment and evoked responses to oscillatory dynamics in auditory cortex remain an active area of investigation (101, 102), converging evidence points to facilitatory effects on perception (97) and memory (103) when endogenous cortical rhythms align with the temporal structure of the acoustic speech signal.

Integrating auditory and motor findings, endogenous theta oscillations may differ in their sensitivity to acoustic input across regions of the speech cortex. Auditory-dominant areas can entrain to rhythmic structure in the incoming speech signal, whereas motor-dominant regions can oscillate independently of acoustics, supporting the intrinsic generation of distributed motor commands. Interactions between these cortical rhythms likely depend on cognitive state: during listening, envelope tracking in auditory cortex may facilitate alignment between perceptual and premotor processes, whereas during production, intrinsic premotor theta may align auditory regions with motor output to optimize acoustic feedback processing. Future work should clarify the relationship between the motor-related rhythm described here and the auditory rhythmicity found during perception, and determine how shared oscillatory dynamics across motor and auditory regions support feedback integration, speech comprehension, and memory (103, 104).

The sensorimotor theta rhythm likely imposes fundamental constraints on the temporal limits of articulation. Recent work has identified a universal upper bound on speech production rate that closely aligns with the upper limit of the theta range (60). Coupé and colleagues (105), for instance, measured syllable rates across 17 languages and found values from 4.3 to 9.1 syllables/sec, averaging 6.63 syllables/sec, with virtually no language exceeding ~7–8 syllables/sec.

Consistently, our EMA analysis showed that even during participants’ fastest speech, AC pulse rates remained below 9 pulses/sec (which depending on the precise proportion of open versus closed syllables (58) corresponds to ~6–8 syllables/sec). These behavioral observations align well with our interpretation that the sensorimotor theta rhythm provides a rhythmic scaffold for coordinating speech movements, with the flexibility to skip cycles as needed (Fig. 6A–C) in order to generate output at rates below the theta range.

Finally, our findings also carry important clinical implications. The theta rhythm we identified may play a central role in speech disorders such as anarthria, apraxia of speech, stuttering, and various forms of aphasia—opening new avenues for therapeutic interventions targeting theta oscillations through neurofeedback or neuromodulation. Furthermore, our demonstration that speech decoding performance peaks around the theta trough has direct relevance for the development of speech neuroprostheses and brain-computer interfaces (106). This mesoscopic LFP rhythm opens a window into the “master clock” of distributed cortical processing—one that may be leveraged to optimize decoding accuracy and improve temporal precision in neural interfaces.

### Conclusion.

Our study establishes sensorimotor theta oscillations as a core temporal organizer of articulatory gestures during fluent speech, opening new avenues for both theoretical inquiry and translational applications. This work sets a clear agenda for future investigations into rhythmic brain mechanisms underlying skilled, dexterous, and coordinated behaviors.

## Supplementary Material

Supplement 1

## Data Availability

The data supporting the findings of this study will be made available upon reasonable request. Requests for materials should be addressed to EFC (Edward.chang@ucsf.edu). The analysis code developed for this study will be made publicly available on GitHub following publication: https://github.com/itziknorman/Norman_et_al_2025_Sensorimotor_Theta
