# Supplementary material for: Sensorimotor Theta Oscillations Coordinate Speech Movements
Source: bioRxiv. 2025 Oct 10:2025.10.09.681482. Preprint. [Version 1] doi: 10.1101/2025.10.09.681482 (PMC12632566; doi:10.1101/2025.10.09.681482)
Supplement: Supplement 1 [file NIHPP2025.10.09.681482v1-supplement-1.pdf]

bioRxiv preprint doi: https://doi.org/10.1101/2025.10.09.681482; this version posted October 10, 2025. The copyright holder for this preprint (which was not certified by peer review) is the author/funder, who has granted bioRxiv a license to display the preprint in perpetuity. It is made available under aCC-BY 4.0 International license.

Supplementary Materials for

Sensorimotor Theta Oscillation Coordinates Speech Movements

Yitzhak Norman[1,2], Loren M. Frank[4,5,6], Edward F. Chang[1,2,3,*]

*Corresponding author: EFC, Edward.Chang@ucsf.edu

The PDF file includes:

Materials and Methods
Figs. S1 to S12
References

bioRxiv preprint doi: https://doi.org/10.1101/2025.10.09.681482; this version posted October 10, 2025. The copyright holder for this preprint (which was not certified by peer review) is the author/funder, who has granted bioRxiv a license to display the preprint in perpetuity. It is made available under aCC-BY 4.0 International license.

Materials and Methods

Participants

Seventeen participants (5 females, 12 males; age range: 22–60 years, M = 36.3) with medication-resistant epilepsy underwent chronic implantation of high-density subdural electrode arrays and depth electrodes as part of their evaluation for neurosurgical treatment (implanted hemisphere: 12 left, 5 right). Electrode locations were determined based on clinical requirements for each patient's respective surgery. No participants had a history of cognitive deficits relevant to the aims of the present study. All participants were fluent in English. Participants provided written informed consent before participating in the studies. All procedures were approved by the University of California, San Francisco Institutional Review Board.

Intracranial data acquisition

Electrocorticographic signals were acquired using subdural high-density grids (Integra or AdTech) with 1.17-mm-diameter exposed contacts and 4-mm inter-electrode spacing. The patients also had depth electrodes implanted in several subcortical regions. The voltage time-series (raw signal) from each electrode contact was amplified and digitized at a sampling rate of 3,051.7578 Hz using a pre-amplifier (PZ5, Tucker-Davis Technologies) and processed through a digital signal processor (RZ2, Tucker-Davis Technologies). During recordings, signals were referenced to the PZ5's internal ground, which operated on battery power. This reference configuration proved remarkably stable, enabling low-noise, reliable recordings without the need to connect a head-mounted local reference electrode (e.g., a subgaleal electrode) to the preamplifier. Given the high signal quality, offline re-referencing was not required, allowing stable, artifact-free **monopolar** acquisition. Speech audio was recorded using a dynamic microphone (e845-S, Sennheiser), amplified by a microphone amplifier (MA3, Tucker-Davis Technologies), and digitized via the same RZ2 digital signal processor.

Electrode anatomical localization

Pre-operative anatomical MRI and post-operative computed tomography (CT) scans were co-registered to determine electrode locations relative to the subject's brain. The pial surface was reconstructed from the pre-operative T1-weighted and FLAIR MRI images using Freesurfer v7.3.2 (*107*). Electrode locations were manually labeled and snapped to the nearest point on the reconstructed cortical mesh. We used SUMA to resample and standardize the cortical surface of each subject (*108*, *109*), enabling visualization of electrodes from different subjects on a common SUMA-standardized cortical template (cvs_avg35_inMNI152). This approach preserves precise node-to-node correspondence across cortical meshes while maintaining the anatomical fidelity of each electrode's position relative to the subject's native gyri and sulci (*110*, *111*). Finally, electrodes were registered to the Desikan-Killiany gyral-based cortical atlas included in Freesurfer (*112*), allowing grouping by anatomical region. Cortical electrodes located more than 5 mm from the cortical ribbon were excluded from further analysis.

Experimental paradigm: main task

Participants (N=14) read aloud 200 sentences from the MOCHA-TIMIT database (*113*) presented in four blocks of 50 sentences each, distributed across multiple days of the hospital stay. Three participants completed only two blocks (100 sentences). Within each block, sentences were shown one at a time on a laptop screen, and participants were instructed to read them aloud at their natural speaking rate following a visual go-cue. A random jitter of 1500 ± 500 ms was inserted between sentence presentation and the go-cue. The order of the sentences was randomized, and trials were advanced manually by the experimenter, allowing a few seconds of rest between consecutive sentences when needed. On average, utterance duration was 3.28 ± 0.61 s. Microphone

bioRxiv preprint doi: https://doi.org/10.1101/2025.10.09.681482; this version posted October 10, 2025. The copyright holder for this preprint (which was not certified by peer review) is the author/funder, who has granted bioRxiv a license to display the preprint in perpetuity. It is made available under aCC-BY 4.0 International license.

recordings were obtained synchronously with the ECoG recordings. Trial onset and go-cue times were tagged using a photodiode and analog triggers sent on the audio channels. The MOCHA-TIMIT database, from which the sentences were drawn, is derived from the TIMIT corpus and designed to cover the full range of phonetic contexts in American English.

Control tasks

Our study includes four key experimental controls: passive listening, silent rest, spontaneous speech production, and silent miming. All fourteen participants who performed the main reading task also completed a passive listening condition, during which they heard 225–600 sentences from the TIMIT acoustic-phonetic corpus (*114*), spoken by 286 male and 116 female speakers from various regions across the United States. Stimuli were presented through free-field speakers using custom MATLAB software on a Windows laptop. Sentences were delivered in pseudorandom order, with a 1 s silent interval between each.

Twelve participants additionally completed an autobiographical interview task, in which they were asked to recount personal experiences or memories; from these recordings, we extracted sentences spanning a broad range of natural, conversational utterances. Nine subjects also performed silent resting-state recordings lasting several minutes. These three conditions were compared to the main sentence-reading task (Fig. S3).

Finally, five subjects performed the silent-miming control task, which involved overtly producing a sentence, followed by a brief pause (~1 s), and then silently miming the same sentence without producing sound (Fig. S2).

Preprocessing and data analysis

Data analysis was performed in MATLAB 2024b (MathWorks Inc., Natick, MA) using EEGLAB v2021.1(*115*), the mTRF toolbox (*66*), Chronux v2.12 (*116*), CircStat toolbox (*117*) and custom-developed code. The raw iEEG signal was statistically inspected to identify and exclude noisy, disconnected, or non-functional channels. Specifically, channels whose voltage values, voltage derivatives, or RMS amplitudes in the top 1% (i.e., the 99th percentile) exceeded 5 standard deviations relative to other electrodes were flagged and subsequently reviewed via visual inspection in both time and frequency domains before exclusion. Preprocessing included notch filtering of the electrocorticographic signal to remove 60 Hz line noise and its harmonics at 120 Hz and 180 Hz, using a zero-lag, linear-phase Hamming windowed FIR band-stop filter (3 Hz wide), followed by resampling to 1000 Hz. Depending on the requirements of the specific analysis, the sampling rate was further downsampled to 500 Hz or 100 Hz.

High-Gamma Activity

In this study, High-gamma (HG) activity—also known as High-Frequency Broadband (HFB) signal—was defined as the mean normalized power across frequencies from 60 to 160 Hz. Neural activity within this range is a well-established electrophysiological marker of local population firing (*118–120*). HG power was computed by: (1) band-pass filtering the ECoG signal in 20 Hz-wide bands (e.g., 60–80, 80–100, etc.) using zero-phase Hamming-windowed FIR filters (10 Hz transition width); (2) extracting the envelope of each narrow band signal by taking the absolute value of the analytic signal obtained from a Hilbert transform; (3) normalizing each amplitude time series by its mean; (4) averaging the normalized envelopes; and (5) multiplying the averaged time series by the mean amplitude across all bands, to restore voltage units. This normalization corrects for the 1/f spectral decay and yields a single broadband amplitude time series per electrode, reflecting local neuronal activity (*110*, *121*).

Identification of speech responsive sites

bioRxiv preprint doi: https://doi.org/10.1101/2025.10.09.681482; this version posted October 10, 2025. The copyright holder for this preprint (which was not certified by peer review) is the author/funder, who has granted bioRxiv a license to display the preprint in perpetuity. It is made available under aCC-BY 4.0 International license.

We identified speech-responsive sites by comparing high-gamma (HG) amplitude during articulation versus the immediate pre-speech (–0.3 to –0.1 s) or post-speech (sentence end + 0.1 to 0.3 s) silent periods, using the Wilcoxon signed-rank test applied individually to each electrode. P-values from all electrodes were pooled to control the false discovery rate (FDR), and electrodes with FDR-corrected $P < 0.05$ in either comparison (relative to the pre- or post-speech window) were classified as speech-responsive. To quantify the magnitude of the response, we also computed the speech-response effect size using the standardized mean difference (Hedges' g; see Fig. S1).

Spectrograms

Depending on the analysis goal, time-frequency decomposition of the ECoG signal was performed using either the fast Fourier transform with a 1-second Hamming-tapered window or a Morlet wavelet transform, as implemented in EEGLAB (*115*). We used a window of 3 cycles at the lowest frequency (4 Hz) with a window scaling factor of 0.8 and a step size of 30–50 ms. Where relevant, spectrograms were normalized by dividing the power at each frequency by the geometric mean power computed over the pre-speech baseline window (−1 to 0 s), followed by conversion to decibels ($10 \times \log_{10}$) and baseline subtraction (*122*). For analyses focusing on the overall shape of the power spectrum (e.g., assessing the presence of a theta-band peak), rather than transient power fluctuations, power at each frequency was divided by the total power across all frequencies, yielding a spectrogram of relative power. To minimize the influence of transient spikes and electrical artifacts on spectral analysis, we inspected the raw signal from each electrode for voltage fluctuations exceeding 3σ relative to all other channels and excluded any trials in which such transients occurred.

Phase Coherence Analyses

Event-related coherograms quantifying phase synchronization between two electrodes (a, b) across n trials were computed using the method described in EEGLAB's 'newcrossf.m' function (*123*):

$$COH^{(a,b)}(f,t) = \frac{1}{n}\sum_{k=1}^{n} \frac{F_k^a(f,t) \cdot \overline{F_k^b(f,t)}}{\left|F_k^a(f,t)\right| \cdot \left|F_k^b(f,t)\right|}$$

where $\overline{F_k^b(f,t)}$ denotes the complex conjugate of $F_k^b(f,t)$. The normalization factor in the denominator ensures that only the relative phase relationship between the two spectral estimates is considered across trials, independent of differences in power. Coherence magnitude ranges from 0 to 1, with 0 indicating a complete absence of phase-synchronization at frequency f in the time window centered at time t, and 1 indicates perfect phase locking. The phase difference between electrodes can then be computed by taking the angle of the complex-valued coherence estimate (across n trials). For consistency across subjects, depth electrodes were excluded from the analysis. To minimize potential contributions from passive volume conduction, pairwise coherence was computed only between speech-responsive grid electrodes separated by at least 15 mm (see Fig. S5A).

Relative theta phase across speech-responsive electrodes

To assess systematic theta-phase differences among speech-responsive electrodes during continuous speech (Fig. S5, S8; Fig. 5) we filtered the raw ECoG signal in the theta range (5–11 Hz) using a zero-lag, linear-phase FIR bandpass filter with a 2 Hz roll-off (Hamming window) and extracted the instantaneous phase using the Hilbert transform. A reference site in the most ventral vSMC was selected based on anatomical location and theta power. For each speech-responsive electrode, relative theta phase was quantified as the mean phase difference from this reference site across artifact-free speech intervals (see above).

bioRxiv preprint doi: https://doi.org/10.1101/2025.10.09.681482; this version posted October 10, 2025. The copyright holder for this preprint (which was not certified by peer review) is the author/funder, who has granted bioRxiv a license to display the preprint in perpetuity. It is made available under aCC-BY 4.0 International license.

Power Spectral Density Analyses

To obtain a robust estimate of the power spectral density (PSD) of the raw ECoG signals, we employed the multitaper method (*124*, *125*), as implemented in Chronux 2.12 (*116*), an open-source MATLAB toolbox. The multitaper approach reduces the variance of spectral estimates by applying multiple orthogonal windowing functions (Slepian tapers) to the data. This produces a set of independent spectral estimates, which are then averaged to yield a more stable and reliable PSD estimate. The continuous intracranial ECoG signal recorded during the task was segmented into 6-second epochs aligned to speech onset (−1 to +5 s). Spectral estimates were computed using five Slepian tapers, yielding a frequency resolution of 1 Hz. Prior to analysis, all data segments were demeaned and zero-padded to a length of 4,096 time points (~8 s).

Detection of Oscillatory Peaks in the Power Spectrum Peak

To detect spectral peaks and assess their prominence, we applied two complementary methods. In Fig. 1C, we computed the power spectral density after first standardizing each voltage time series by rescaling its values between the 1st and 99th percentiles. This normalization ensured a comparable dynamic range across electrodes, allowing consistent comparison and visualization of spectral profiles while minimizing bias from inter-electrode amplitude differences. The multitaper power spectrum was then computed on the rescaled signals. To isolate spectral peaks, we removed the 1/f background by fitting a linear regression to the 2–30 Hz range and removing the fitted trend. The resulting spectrum was z-scored, smoothed using a Savitzky–Golay filter (2nd-order polynomial, 1 Hz window; 'sgolayfilt.m'), and subjected to peak detection. Peak prominence was defined as the height of a peak relative to its surrounding baseline, expressed in z-scored power values.

In all other analyses of spectral peaks, we applied a parametric power spectrum decomposition using the FOOOF algorithm (*48*) which transforms power to a log-scale, separates the aperiodic 1/f component from the oscillatory components and then applies peak detection on the 1/f corrected spectra. We used the default FOOOF parameters (2–30 Hz frequency range; peak threshold = 2.5) and excluded peaks with widths > 6 Hz. When comparing across different tasks (e.g., Fig. S3), we applied the voltage-rescaling procedure described above prior to power spectrum estimation and FOOOF analysis. When comparing across speech rates (Fig. 2A-C), we skipped this step, as the comparison is done within electrode.

Quantifying Phase-Amplitude Coupling (PAC)

Phase-amplitude coupling (PAC) was measured using Tort's Modulation Index (MI) approach (*56*, *57*). Instantaneous theta phase was extracted via the Hilbert transform and divided into 24 equally spaced bins spanning the full $[0, 2\pi]$ range. High-gamma (HG) amplitude was averaged within each phase bin during speech intervals, excluding pre- and post-utterance silent periods. The resulting phase–amplitude distribution was compared to a uniform distribution using Kullback–Leibler divergence to quantify the degree of modulation. This yielded an MI value reflecting the extent to which HG amplitude was modulated by theta phase. PAC was computed separately for each electrode, and statistical significance was assessed by comparing the observed MI to a surrogate distribution generated from 5,000 random circular shifts of the theta-phase time series. To quantify coupling between theta phase and articulatory change (AC), we applied the same procedure, this time using the AC time series. To ensure comparability across electrodes and subjects, both HG and AC time series were rescaled to the [0, 1] range prior to computing the modulation index, allowing the analysis to focus on phase coupling independent of absolute amplitude differences. To generate the comodulogram shown in Fig. S6, we computed the MI across multiple narrowband frequency pairs: phase frequencies were filtered using a fixed 4 Hz bandwidth in 1 Hz steps from 4 to 20 Hz, and amplitude frequencies were log-spaced from 20 to

bioRxiv preprint doi: https://doi.org/10.1101/2025.10.09.681482; this version posted October 10, 2025. The copyright holder for this preprint (which was not certified by peer review) is the author/funder, who has granted bioRxiv a license to display the preprint in perpetuity. It is made available under aCC-BY 4.0 International license.

160 Hz, with bandwidths increasing from 4 Hz at the lowest frequency to 40 Hz at the highest. For consistency across subjects, only speech-responsive electrodes located on the cortical grid were included in this analysis; depth electrodes were excluded.

Acoustic-to-Articulatory Inversion (AAI) and Tract Variables

Many critical vocal tract movements involved in speech production are not externally visible and cannot be easily monitored. Capturing the kinematic trajectories of articulators such as the jaw, lips, and tongue requires imaging techniques capable of dynamically tracking both external and internal articulatory motion during continuous speech. One such method is electromagnetic midsagittal articulography (EMA), which uses sensors placed on key points of the vocal tract to monitor their movement in real time within a magnetic field during fluent speech.

However, because simultaneous acquisition of EMA and ECoG data is not practically feasible, we employed a deep-learning-based method to infer articulatory dynamics directly from the acoustic signals recorded via microphone (*5*, *61*). This state-of-the-art acoustic-to-articulatory inversion (AAI) technique operates in the *tract-variable* space—a set of parameters derived from the trajectories of the tracked EMA sensors, that quantify the degree and location of vocal-tract constrictions. These variables capture key geometric distances along the vocal tract that define each constriction gesture. This representation yields a speaker-independent, gesture-relevant model of articulatory behavior (*3*, *126*) and enables accurate AAI-based reconstruction of articulatory kinematics that generalizes well to unseen speakers (*61*).

Using the AAI technique, we monitored the kinematic trajectories of 9 tract-variables at 100 Hz temporal resolution: LA, lip aperture; LP, lip protrusion; TBCL, tongue body constriction location; TBCD, tongue body constriction degree; TTCL, tongue tip constriction location; TTCD, tongue tip constriction degree; TRCL, tongue root constriction location; TRCD, tongue root constriction degree; and JA, jaw angle.

The geometric transformations used to compute tract-variables from EMA sensor positions are detailed in (*61*, *127*). We applied these transformations to raw EMA measurements from the Haskins Rate Production Comparison (HPRC) database (*128*) and derived tract-variable trajectories representing ground-truth articulatory kinematics. These were analyzed in parallel with the AAI-inferred trajectories obtained from our intracranially monitored ECoG participants (Fig. 3). This comparison served to validate the AAI reconstructions and to ensure that our characterization of vocal tract kinematics using the Articulatory Change (AC) feature was not biased by potential inaccuracies in the inversion process.

Articulatory Change

To capture the dynamics of articulatory change over time—that is, the temporal modulation function of the articulatory process—we adapted a method recently proposed by Goldstein (*58*) to our nine EMA-derived tract-variables. This method quantifies articulatory change as the sum of squared velocities across tract-variables:

$$AC(k) = \sum_{i=1}^{9} (m(i, k+1) - m(i, \mathrm{k}))^2$$

where $m(i,k)$ denotes the value of the i-th tract variable at frame k. This univariate signal reflects the instantaneous magnitude of articulatory movement across the vocal tract and serves as a proxy for the temporal modulation of articulatory gestures during continuous speech. To align the AC time series with the sampling rate of the ECoG signal, we first computed the tract-variable derivatives at its original 100 Hz sampling rate, then upsampled the resulting time series to the target resolution using spline interpolation.

Articulatory Change Pulse Rate

bioRxiv preprint doi: https://doi.org/10.1101/2025.10.09.681482; this version posted October 10, 2025. The copyright holder for this preprint (which was not certified by peer review) is the author/funder, who has granted bioRxiv a license to display the preprint in perpetuity. It is made available under aCC-BY 4.0 International license.

To compute the rate of AC pulses during continuous speech while maintaining consistent detection criteria across subjects, we first applied peak detection to sentence-level AC traces from the EMA dataset. Utterances with syllable rates below the 1st percentile or above the 99th percentile across all sentences, or those containing articulatory pauses longer than 0.5 seconds, were considered outliers and excluded. AC pulses were then identified using MATLAB's findpeaks.m function, with a minimum peak distance of 20 ms. From the detected peaks, we derived group-level distributions of peak prominence and width values, pooled across both normal and fast speech. The 1st percentile of each distribution was selected as the threshold for subsequent peak detection. These empirically derived thresholds were then applied to both the EMA and ECoG datasets, ensuring uniform and data-driven pulse detection across all subjects and conditions. In all subsequent analyses, excessively slow sentences—defined as those with syllable rates falling below the first quartile minus 1.5 times the interquartile range, or those containing articulatory pauses longer than 0.5 seconds—were excluded.

Phonetic and phonological transcription

Transcriptions of the recorded speech acoustics began with automatic speech-to-text transcription using Adobe Premiere Pro 5, followed by manual correction at the word level, ensuring that the transcript reflected the vocalization that the participant actually produced. Using these sentence-level transcriptions and the corresponding acoustic utterances, we performed sub-phonetic alignment following previously described methods (*129*, *130*), and extracted the onsets of individual phonemes, including vowels constituting syllabic nuclei. To ensure a stable and standardized method for counting syllables per utterance, we used the Datamuse API (https://www.datamuse.com/api) to retrieve dictionary-based syllabification for each produced word or its closest phonological match, based on American English pronunciation.

Speech errors

During transcription, utterances containing speech errors that resulted in significant fluency disruptions or were immediately followed by a speech repair were logged and excluded from subsequent analyses. These error trials were then specifically analyzed in Fig. S11. Typical examples included substitutions or mid-word stopping followed by immediate repair, such as: *"Curiosity and media(*) mediocrity seldom coexist"* or *"Publice(*) publicity and notoriety go hand in hand"*. The error trial rate across participants was 7.5 ± 5.8% of sentences.

Multivariate temporal response function (mTRF) encoding models

We used the mTRF toolbox (*66*) to fit multivariate encoding models using ridge regression (*5*, *131*, *132*). For a detailed mathematical description of the fitting procedure, see the original reference of the toolbox. Regularization strength (λ) was optimized using 10-fold cross-validation on a training set comprising 90% of the data. Model performance was evaluated on the remaining 10% (held-out test set) by computing the Person's correlation (r) between the predicted and actual signals.

This framework was applied in two analyses. First, to recover the temporal profile of articulatory change (AC) during the production of individual phonemes and syllables (Fig. 3F), we modeled the AC time series as a linear sum of overlapping event-related responses time-locked to phoneme onsets. Phonemes were grouped into six classes: stops, fricatives, affricates, nasals, liquids, and syllabics. Both the phonemic predictors and AC responses were z-scored within each trial. The model was trained on data resampled to 100 Hz, with time lags from –1250 to +1250 ms. On average, the fitted models—validated on a held-out test set—achieved an r value of 0.37 ± 0.05 (mean ± s.d) up to r=0.5.

In the second analysis, we modeled the HG amplitude time series at each electrode as a weighted sum of articulatory kinematic features over time. These features—derived from AAI-

bioRxiv preprint doi: https://doi.org/10.1101/2025.10.09.681482; this version posted October 10, 2025. The copyright holder for this preprint (which was not certified by peer review) is the author/funder, who has granted bioRxiv a license to display the preprint in perpetuity. It is made available under aCC-BY 4.0 International license.

extracted tract variables (Fig. 3)—captured movements of the jaw, lips, and tongue. The model included the instantaneous position of the articulators (constriction degree and location; 9 features), their velocity (first derivative; 9 features), acceleration (second derivative; 9 features), and their overall speed—i.e., the Articulatory Change (AC) time series—totaling 28 features. To ensure comparability across features and trials, both the kinematic predictors and HG responses were z-scored within each trial (i.e., centered and normalized). The model was trained on data resampled to 100 Hz, with time lags from –1050 to +1050 ms. In electrodes where theta phase was significantly coupled to AC, the fitted models—validated on a held-out test set—showed r value of 0.22 ± 0.13 (mean ± s.d), up to r=0.54, consistent with our previously reported estimates in vSMC (*5*). To determine whether the AC feature itself accounted for a significant amount of unique variance, we repeated the modeling procedure using a temporally shuffled version of the AC predictor. Model performance (r^2) was then compared between the original and shuffled models across 200 iterations (see Fig. S9A). Statistical significance was assessed by calculating the proportion of iterations in which the shuffled model's performance equaled or exceeded that of the original model.

State space analysis

To investigate the relationship between theta phase and the mesoscopic SMC activation elicited during articulatory movements, we projected multi-electrode HG activity patterns onto a two-dimensional state space (*1*). This projection was computed by applying principal component analysis (PCA) to z-scored peri-AC HG activation profiles derived from the mTRF encoding model, including only SMC electrodes that showed significant theta-movement coupling. The first ten components accounted for 64.6% of the total variance. HG activity was then mapped onto the first two principal components (explaining 33% of the variance) and color-coded according to the circular mean of the relative theta phases of the most active electrodes at each point along the trajectory—defined as those with the top 10% of PC_1 and PC_2 scores.

Phase-aligned averaging of peri-syllable articulatory change

To investigate the relationship between consecutive theta cycles and AC during sequential gestures grouped within a syllable, we developed a method that enables averaging across events of interest—i.e., syllabic nuclei—while preserving multi-cycle phase alignment between the ongoing theta oscillation and the concurrent AC signal (Fig. 6A, Fig. S10). Theta-band–filtered ECoG and AC time series were epoched from –750 to 750 ms relative to the syllable nucleus (i.e., vowel onset). Only syllables that were temporally isolated—preceded by at least 250 ms of articulatory pause—were included. Articulatory pauses were defined as periods without detectable AC pulses, identified using empirically determined peak prominence and width thresholds, based on actual EMA recordings from the Haskins Production Rate Comparison (HPRC) database (see the *Articulatory Change Pulse Rate* subsection for details). Syllables with extreme durations (outside the 1st and 99th percentiles) were excluded, yielding several hundred valid syllables per subject. For simplicity, analyses were restricted to a single sensorimotor electrode per subject—specifically, the one exhibiting the strongest theta–AC coupling.

To preserve phase alignment across syllables, each epoch was aligned to the theta trough closest to the syllable nucleus, and the unwrapped analytic theta phase was extracted. Continuous phase series were then resampled via linear interpolation onto a uniform phase axis spanning eight consecutive theta cycles (from -9π to 7π relative to the nucleus-aligned trough). The same interpolation was applied to the AC signal, enabling across-syllable averaging while maintaining precise phase alignment with the theta cycle—thus avoiding phase mixing or cancellation.

In the group-level analysis, phase-aligned AC profiles were averaged across subjects after re-aligning each electrode to the phase of maximal AC in that electrode, as determined in Fig. 4E. To

bioRxiv preprint doi: https://doi.org/10.1101/2025.10.09.681482; this version posted October 10, 2025. The copyright holder for this preprint (which was not certified by peer review) is the author/funder, who has granted bioRxiv a license to display the preprint in perpetuity. It is made available under aCC-BY 4.0 International license.

assess the statistical significance of AC modulation by theta phase, the actual observed pattern was compared to a surrogate distribution generated by circularly shifting the theta phase by a random amount and repeating the full analysis 50 times. Because both real and shuffled analyses centered epochs on the trough closest to the syllable nucleus, this procedure preserved slower dynamics associated with syllable production while disrupting only the fine-grained theta phase relationship. Contrasting the observed and shuffled profiles thus isolated the specific contribution of theta phase to articulatory change during syllables. We quantified this contrast as the percent change between the observed and shuffled profiles for each electrode and tested it against zero (N=14, one electrode per subject) using Wilcoxon signed-rank tests (Fig S10). P-values were corrected for multiple comparisons across phase bins using FDR.

Phase-aligned syllable decoding analysis

To determine whether articulatory information encoded at the mesoscopic scale emerges transiently at specific phases of the theta cycle, we trained a sliding-window syllable decoder that advanced along the unwrapped theta phase axis, rather than time. We selected the 12 most common syllables in our dataset (e.g., /ri/, /ɪz/, /fɔr/, /lɪ/, /ju/)—defined as those with at least 10 repetitions across sentences, sufficient to train a classifier. The HG time series was aligned across trials using the procedure described above, centering each data epoch on the theta trough closest to the syllable nucleus. Electrodes exhibiting robust theta–movement coupling (285 sites across 10 subjects) were pooled to construct a group-level matrix of HG activity during syllable production (electrodes × phase × trials). As in the analysis in Fig 6A-C, activity in each trial was aligned to a common phase axis spanning eight consecutive theta cycles (from -9π to 7π relative to the nucleus-aligned trough). This phase alignment enabled decoding performance to be tracked as a function of instantaneous theta phase, rather than time, across multiple consecutive cycles (Fig. 6D-F).

For the decoding analysis, phase-aligned HG activity patterns were z-scored across trials and re-binned into 0.8-radian-wide phase bins with a 0.3-radian step size, ensuring a fixed phase resolution across electrodes. To account for the systematic phase differences observed along the dorsoventral axis of the SMC, electrodes were re-aligned to the phase of articulatory movement (i.e., the phase of maximal articulatory change (AC) in each electrode, as determined by the analysis described in Fig. 4E). This step ensured that signals were not only phase-aligned across trials within each electrode—but also centered on a common physiological reference across electrodes: the phase of maximal articulatory movement nearest the syllable nucleus.

A multi-class SVM classifier was trained to decode syllable identity from these phase-aligned HG activity patterns using 5-fold stratified cross-validation, repeated independently at each phase bin (using MATLAB functions fitcecoc.m and cvpartition.m). Decoding accuracy was evaluated on held-out test folds, and the analysis was repeated 25 times with different initializations to obtain a stable estimate of decoding accuracy. To assess statistical significance, a null distribution was generated by repeating the entire decoding procedure 500 times using shuffled trial labels. Crucially, the same permutation was applied across all phase bins within each shuffle iteration, preserving temporal structure while disrupting the syllable label mapping. This allowed fair comparison between real and shuffled decoding performance. Accuracy was smoothed using a 5-point Savitzky–Golay filter (2nd-order polynomial). P-values were computed as the proportion of shuffles in which accuracy exceeded or equaled the mean accuracy of the real data and were corrected for multiple comparisons using FDR.

Statistical tests

All statistical analyses were performed in MATLAB. Unless otherwise noted, pairwise comparisons were conducted using two-sided Wilcoxon signed-rank or rank-sum tests. For circular data, we used the CircStat toolbox (*117*). The unit of analysis was typically individual

bioRxiv preprint doi: https://doi.org/10.1101/2025.10.09.681482; this version posted October 10, 2025. The copyright holder for this preprint (which was not certified by peer review) is the author/funder, who has granted bioRxiv a license to display the preprint in perpetuity. It is made available under aCC-BY 4.0 International license.

subjects, or electrodes nested within subject when using mixed-effects analyses. In some cases, as stated in the main text, the unit was individual electrodes or activity patterns across pooled electrodes (as in the syllable decoding analysis). Resampling tests were performed using custom MATLAB scripts based on previously published routines (*133*, *134*), or with algorithms from the Mass Univariate ERP Toolbox (*135*). Multiple comparisons across electrodes or time bins were corrected using false discovery rate (FDR) adjustment (*136*). Data collection was conducted blind to experimental conditions; data analysis was not. No statistical methods were used to predetermine sample sizes, but sample sizes were consistent with those commonly used in the field (*68*, *111*).

Mixed effects analysis

Mixed-effects analyses were conducted in MATLAB using the 'fitlme.m' function. Models included the relevant fixed effects and a random intercept for 'Subject', with 'Electrode' nested within 'Subject' (or 'Electrode-pair' nested within 'Subject', as in the pairwise coherence analysis). For example, to test for differences in central frequency across speech rates (fixed factor *condition* with three levels—slow, medium, fast), we used the following mixed-effects model:

$$Frequency \sim 1 + condition + (1 + condition|subject) + (1|subject{:}\,electrode)$$

This nesting of electrodes within subjects accounts for the hierarchical structure of the data—specifically, that each participant contributed multiple electrodes (or electrode pairs) to the analysis, and that observations from the same subject or electrode are not statistically independent. It also captures variability arising from the fact that participants contributed different numbers of electrodes. Random slopes were included when justified by the data structure, provided they did not lead to over-parameterization or model non-identifiability. Main effects were tested using Type III ANOVA implemented in 'fitlme.m'. Degrees of freedom were estimated using the Satterthwaite approximation. For analyses involving timepoint-by-timepoint mixed-effects models, p-values were computed individually at each time point and corrected for multiple comparisons using FDR.

bioRxiv preprint doi: https://doi.org/10.1101/2025.10.09.681482; this version posted October 10, 2025. The copyright holder for this preprint (which was not certified by peer review) is the author/funder, who has granted bioRxiv a license to display the preprint in perpetuity. It is made available under aCC-BY 4.0 International license.

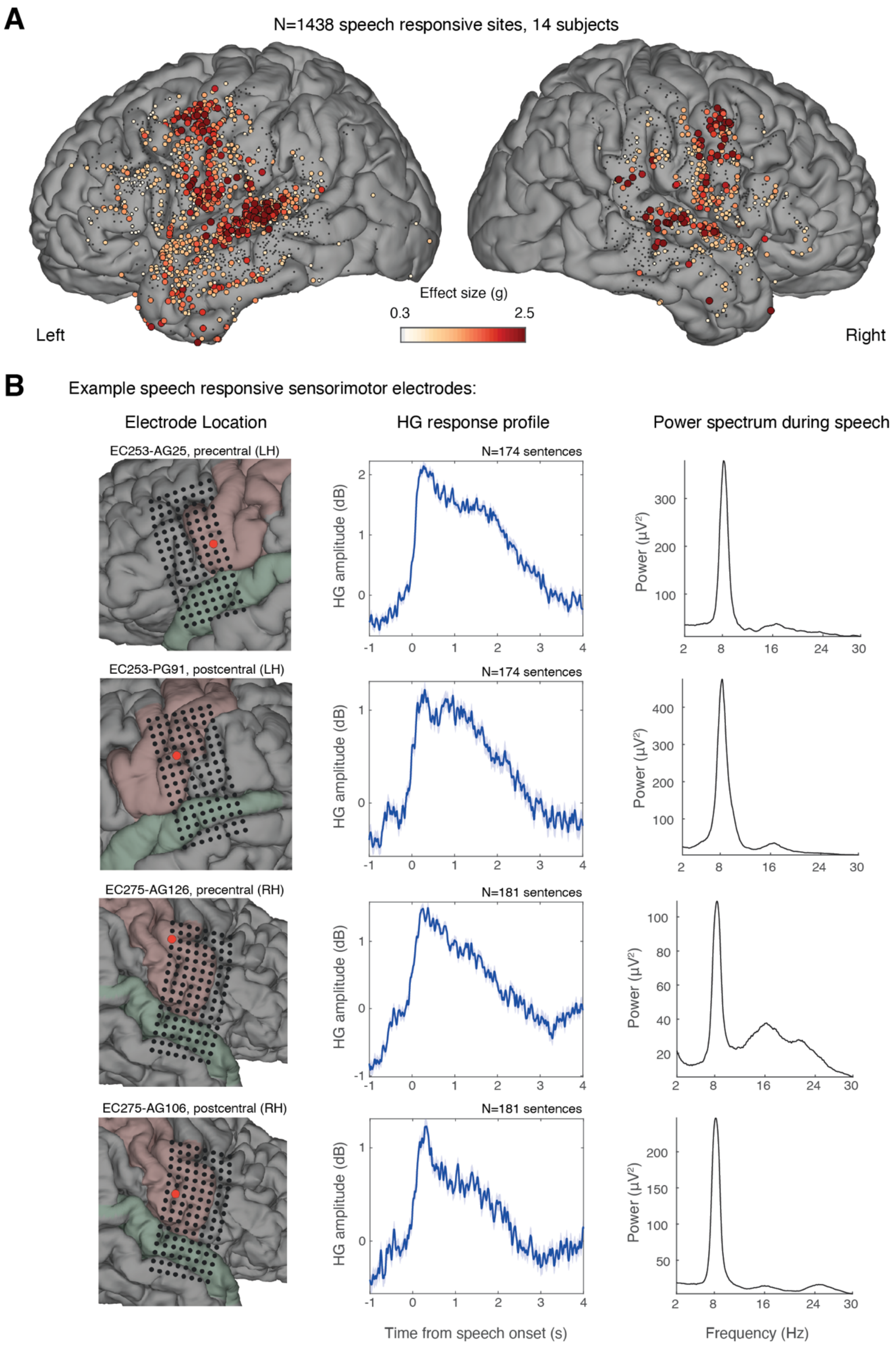

Fig. S1. Overview of speech responsive electrodes.

bioRxiv preprint doi: https://doi.org/10.1101/2025.10.09.681482; this version posted October 10, 2025. The copyright holder for this preprint (which was not certified by peer review) is the author/funder, who has granted bioRxiv a license to display the preprint in perpetuity. It is made available under aCC-BY 4.0 International license.

(A) Anatomical distribution of speech responsive electrodes. Out of 3,599 recording sites analyzed, 1,438 were classified as speech-responsive, showing a marked increase in HG amplitude during speech compared to the inter-trail silent periods ($P < 0.05$, FDR corrected). The spatial distribution of these speech-responsive sites highlights the key cortical regions involved in speech production, including the sensorimotor cortex (SMC), superior temporal gyrus (STG), supramarginal gyrus (SMG), and additional sites in the prefrontal and temporal cortices. We used this gross functional classification of electrodes to focus subsequent analyses on speech-responsive sites, effectively excluding task-irrelevant sites. **(B)** Example of speech-responsive SMC electrodes from two patients. The left panels show the anatomical location of the electrode, the middle panels display HG activity (60-160 Hz) during speech, and the right panels present the multi-taper power spectrum computed over an interval of -1 to 5 s relative to sentence onset, showing a prominent 8 Hz LFP oscillation occurring alongside the HG activity associated with speech production.

bioRxiv preprint doi: https://doi.org/10.1101/2025.10.09.681482; this version posted October 10, 2025. The copyright holder for this preprint (which was not certified by peer review) is the author/funder, who has granted bioRxiv a license to display the preprint in perpetuity. It is made available under aCC-BY 4.0 International license.

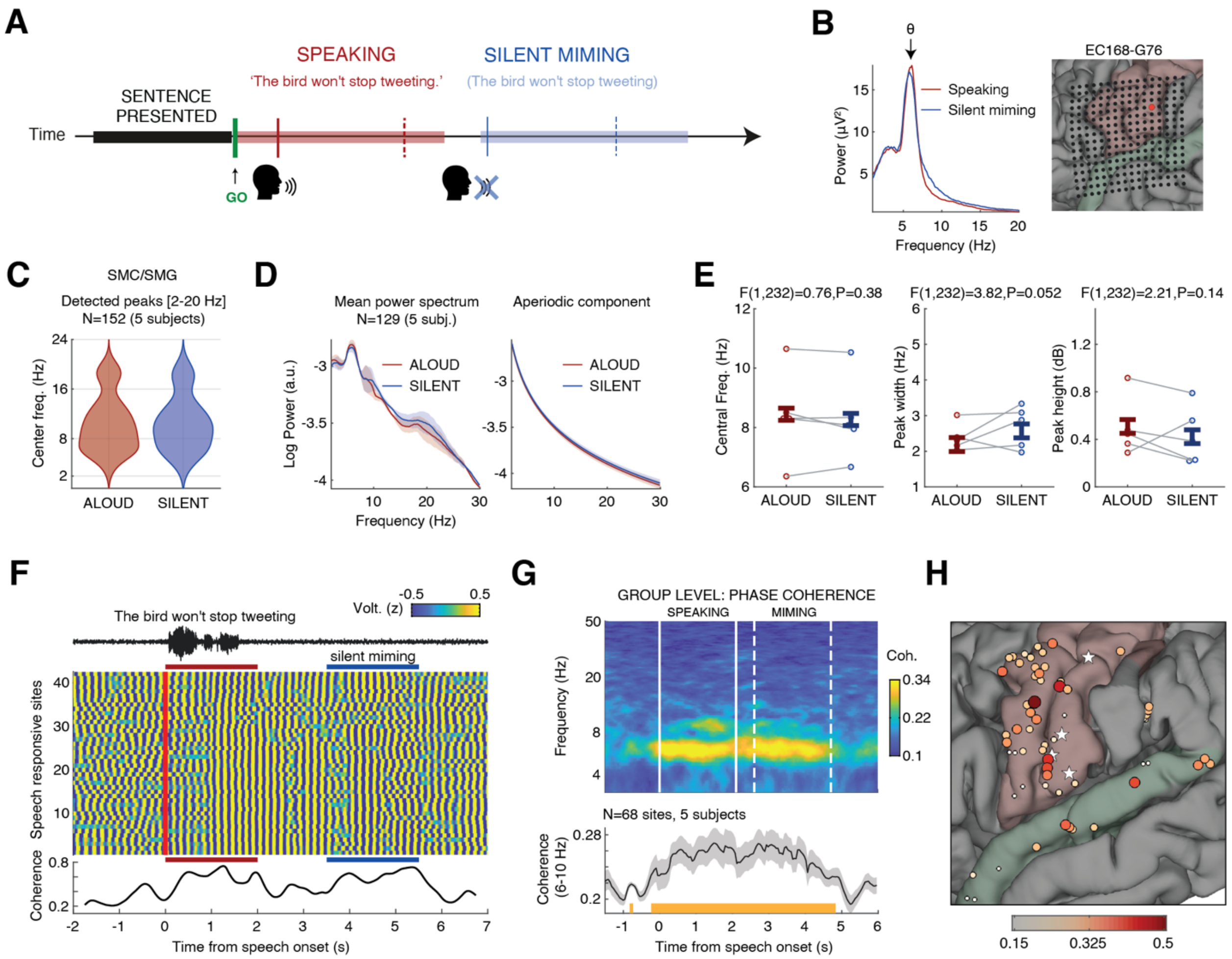

Figure S2. Coherent sensorimotor theta rhythm persists during both overt speech and silent miming.

(A) Five subjects performed a control task that involved overtly producing a sentence, followed by a brief pause (~1 s), and then silently miming the same sentence. (B) Power spectral density in a representative postcentral electrode during a 3.5 s window centered on overt speaking or silent miming. The location of the electrode is shown on the right (red dot). (C) Distribution of spectral peaks (2–20 Hz) detected in speech responsive electrodes across SMC and SMG (N = 152, 5 subjects). Spectral decomposition and peak detection were performed using the FOOOF algorithm (*48*). (D) Left: Mean power spectrum across N=129 SMC/SMG sites that exhibited peaks within the broad theta range (5-11 Hz) showing similar overall shape across the two conditions and comparable aperiodic components. Right: Corresponding aperiodic 1/f component recovered by FOOOF. (E) Direct comparison of the central frequency, width, and height of the detected theta peaks between speaking and miming revealed no significant differences (mixed-effects analysis; p-values shown in figure; error bars represent group mean ± 95% CI). This provides direct evidence that the sensorimotor theta rhythm is not a byproduct of auditory feedback: during miming, participants moved their articulators without producing sound, yet the oscillation retained stable spectral properties comparable to overt speech. (F) Example trial showing momentary increases in theta phase coherence during both speaking (red) and silent miming (blue) across 42 speech-responsive sites in a representative subject (15 traces plotted). Voice amplitude is shown above. (G) Averaged cohereogram across N=68 pairs of speech-responsive sites from 5 subjects, demonstrating a stable increase in theta phase coherence during both speaking and silent miming. Shaded areas: 95% CI of within-electrode differences obtained from the mixed-effects analysis. (H) Spatial distribution of analyzed electrodes, color-coded by coherence during overt speech. White stars mark the reference theta sites used for phase-coherence calculation. Right-hemisphere sites are not shown.

bioRxiv preprint doi: https://doi.org/10.1101/2025.10.09.681482; this version posted October 10, 2025. The copyright holder for this preprint (which was not certified by peer review) is the author/funder, who has granted bioRxiv a license to display the preprint in perpetuity. It is made available under aCC-BY 4.0 International license.

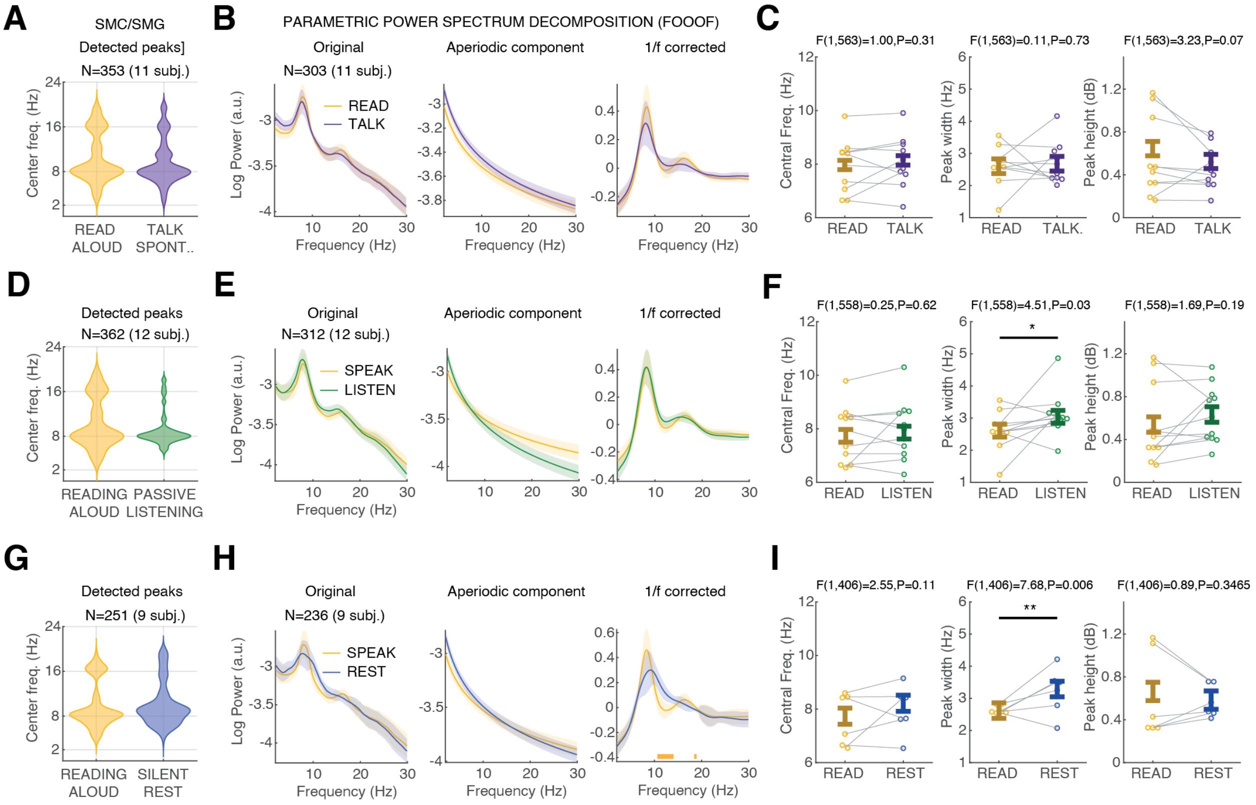

Figure S3. SMC theta oscillations show stability across behavioral states.

All the participants (N=14) completed a control condition in which they passively listened to 225–600 sentences from the TIMIT corpus (*114*). In addition, 12 participants completed an autobiographical interview task, where they were asked to recount a personal experience or memory; from these interviews we extracted sentences spanning a broad range of natural conversational utterances. These two conditions were compared to our main sentence reading task.

(A-C) Comparing reading aloud vs. spontaneous conversational utterances.

(D-F) Comparing reading aloud vs. passive listening.

(G-I) Comparing reading aloud vs. silent rest.

(A,D,G) Distribution of spectral peaks (2–20 Hz) detected in speech responsive electrodes across SMC and SMG. Spectral decomposition and peak detection were performed using the FOOOF algorithm (*48*). **(B,E,H)** Average power spectra across electrodes exhibiting peaks within the theta range (5–11 Hz), with aperiodic and oscillatory components shown separately. Spectra were computed over –0.5 to 3.5 s windows aligned to utterance onset, revealing stable low-frequency profiles (1–30 Hz) across conditions and a prominent oscillatory peak in the theta range. ECoG time series were rescaled prior to spectral decomposition to standardize voltage values across electrodes (see Methods). **(C,F,I)** Mixed-effects analysis comparing the central frequency, width, and height of theta-range peaks. While peak frequency and magnitude remained stable, peaks were significantly broader during passive listening and silent rest (i.e., idle states of the speech motor cortex). Circles indicate subject means; error bars represent group mean ± 95% CI; p-values shown at top.

bioRxiv preprint doi: https://doi.org/10.1101/2025.10.09.681482; this version posted October 10, 2025. The copyright holder for this preprint (which was not certified by peer review) is the author/funder, who has granted bioRxiv a license to display the preprint in perpetuity. It is made available under aCC-BY 4.0 International license.

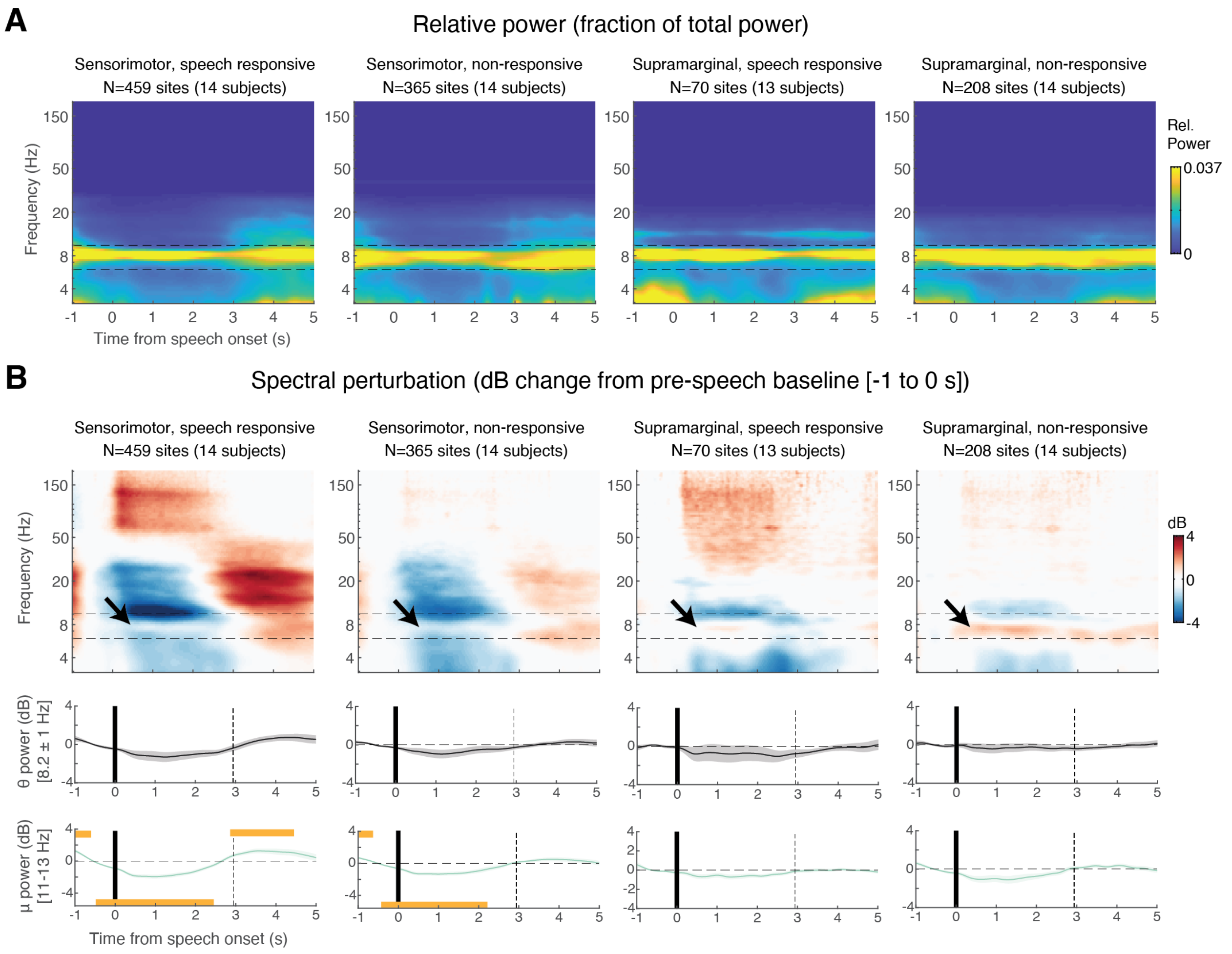

Fig. S4. Wavelet spectrogram aligned to speech onset in SMC and SMG

Morlet wavelet spectrogram, time-locked to speech onset, showing relative power in panel **(A)** and baseline-corrected normalized power in panel **(B)**. In panel (A), colors represent the fractional power at each frequency relative to the total signal power. Notably, there is persistent oscillatory power concentrated at 8 Hz during speech, extending both before and after the utterance (mean sentence duration: 3 ± 0.4 sec). In panel (B), colors represent the dB power change relative to the immediate pre-speech baseline (-1 to 0 s). A timepoint-by-timepoint mixed-effects analysis, comparing the intercept against zero (FDR corrected), revealed no significant power changes at 8 Hz relative to speech onset (middle panels), emphasizing the persistence of the sensorimotor theta oscillation. This stability contrasts sharply with the significant post-speech power decrease in the neighboring Mu band (10–13 Hz), as indicated by the horizontal orange bars on the x-axis marking significant time points.

bioRxiv preprint doi: https://doi.org/10.1101/2025.10.09.681482; this version posted October 10, 2025. The copyright holder for this preprint (which was not certified by peer review) is the author/funder, who has granted bioRxiv a license to display the preprint in perpetuity. It is made available under aCC-BY 4.0 International license.

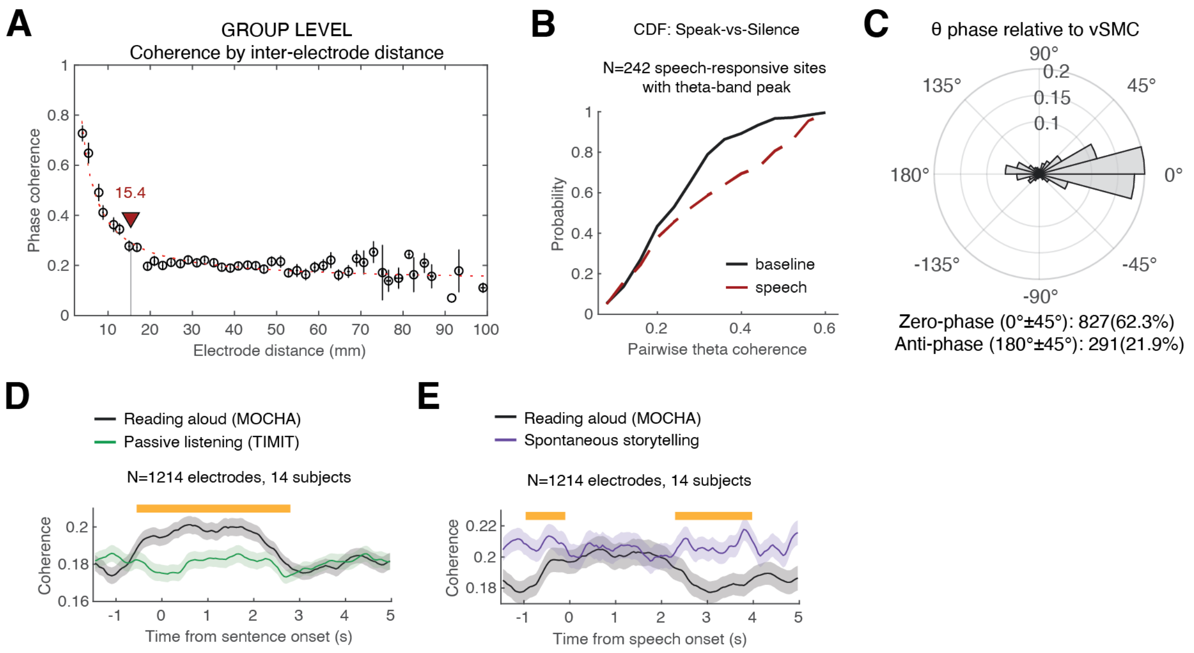

Fig. S5. Phase coherence among speech responsive site across behavioral states.

(A) Mean phase coherence in the theta band (6-10 Hz), computed within a time window of 0 to 4 seconds relative to speech onset, plotted as a function of inter-electrode distance (mm). Electrodes in close proximity exhibited enhanced coherence, potentially attributable to passive conductance effects. For the analysis presented in Fig. 2, we included only electrode pairs separated by at least 15 mm (where the plateau begins) to mitigate this effect.

(B) Cumulative distribution of theta-band phase coherence during speech versus the pre-speech baseline across speech-responsive electrodes exhibiting a detectable theta peak.

(C) Distribution of phase difference across speech responsive sites, relative to a single most ventral SMC speech-responsive electrode in each patient. Most electrodes exhibited near 0-phase coupling (0 ± 45°: 770/1328; 58%). Another substantial portion showed antiphase coupling (180 ± 45°: 277 electrodes; 21%).

(D) Comparing theta phase coherence during speech production and passive listening revealed significantly higher coherence during production, arguing against an auditory-driven account and highlighting its motor-related origin.

(E) Theta phase coherence during reading and spontaneous articulation showed comparable magnitudes, though spontaneous speech exhibited sustained coherence enhancement, consistent with continuous storytelling.

Shaded areas: 95% CI of within-electrode differences obtained from the mixed-effects analysis. Horizontal orange markers indicate statistically significant time points.

bioRxiv preprint doi: https://doi.org/10.1101/2025.10.09.681482; this version posted October 10, 2025. The copyright holder for this preprint (which was not certified by peer review) is the author/funder, who has granted bioRxiv a license to display the preprint in perpetuity. It is made available under aCC-BY 4.0 International license.

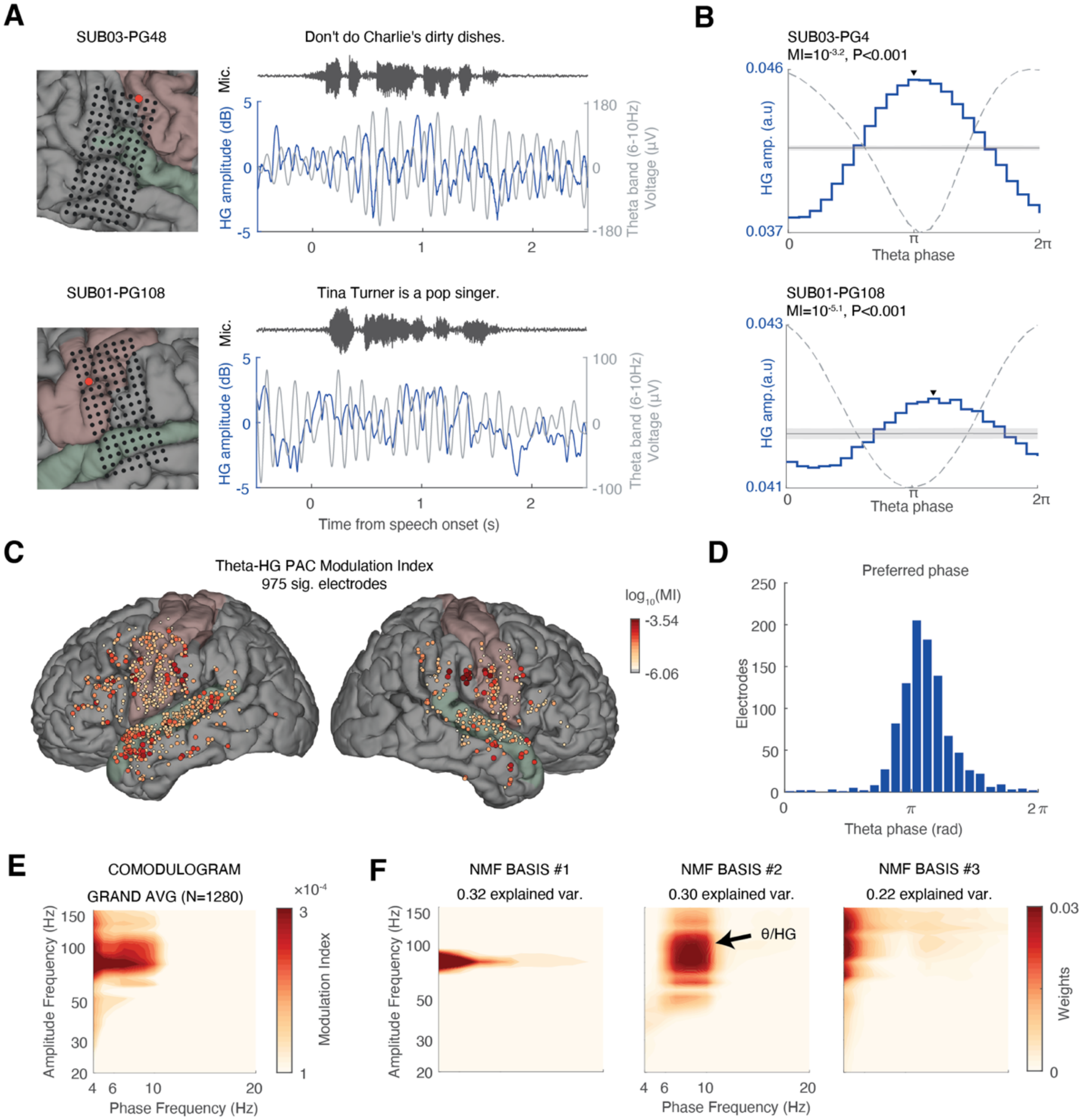

Fig. S6. Theta-HG phase-amplitude coupling (PAC) during speech

(A) Example of speech-responsive vSMC electrodes from two subjects, demonstrating robust theta oscillations and phase-dependent modulation of HG power during articulation. **(B)** To quantify PAC during speech production, we used Tort's Modulation Index (MI), as proposed by (*56*, *57*). The instantaneous theta phase was extracted via a Hilbert transform and binned into 24 phase bins spanning the range [0, 2π]. We then averaged the HG amplitude in each phase bin across all speech intervals and quantified deviations from a uniform distribution using Kullback-Leibler divergence. To statistically assess theta-HG coupling, we compared the theta-HG MI for each electrode to a surrogate distribution generated by circularly shifting the theta phase time series by a random amount 5,000 times (gray horizontal line represent the mean ± SD of the shaffled data). Significance was determined by the proportion of iterations in which the $MI_{shuff.}$ was equal to or greater than the empirical MI. We found that 72.1% (975/1,280) of all speech-responsive electrodes exhibited statistically significant theta-HG coupling (P<0.05, FDR corrected). **(C)** Spatial distribution of MI values across significant electrodes, highlighting the widespread nature of theta-HG PAC. **(D)** Distribution of preferred theta phases across significant electrodes, showing that HG amplitude was consistently

bioRxiv preprint doi: https://doi.org/10.1101/2025.10.09.681482; this version posted October 10, 2025. The copyright holder for this preprint (which was not certified by peer review) is the author/funder, who has granted bioRxiv a license to display the preprint in perpetuity. It is made available under aCC-BY 4.0 International license.

higher near the theta trough. **(E)** Phase-amplitude comodulogram compute using the MI method, averaged across all speech-responsive electrodes (N = 1,280). **(F)** To decompose the grand-average comodulogram into its dominant components, we applied non-negative matrix factorization (NMF) using MATLAB's nnmf.m function. This analysis revealed three dominant PAC components collectively accounting for over 84% of the variation across electrodes. Notably, NMF basis #2 identified a distinct PAC between theta (6–10 Hz) and HG (70–120 Hz), explaining 30% of the variance.

bioRxiv preprint doi: https://doi.org/10.1101/2025.10.09.681482; this version posted October 10, 2025. The copyright holder for this preprint (which was not certified by peer review) is the author/funder, who has granted bioRxiv a license to display the preprint in perpetuity. It is made available under aCC-BY 4.0 International license.

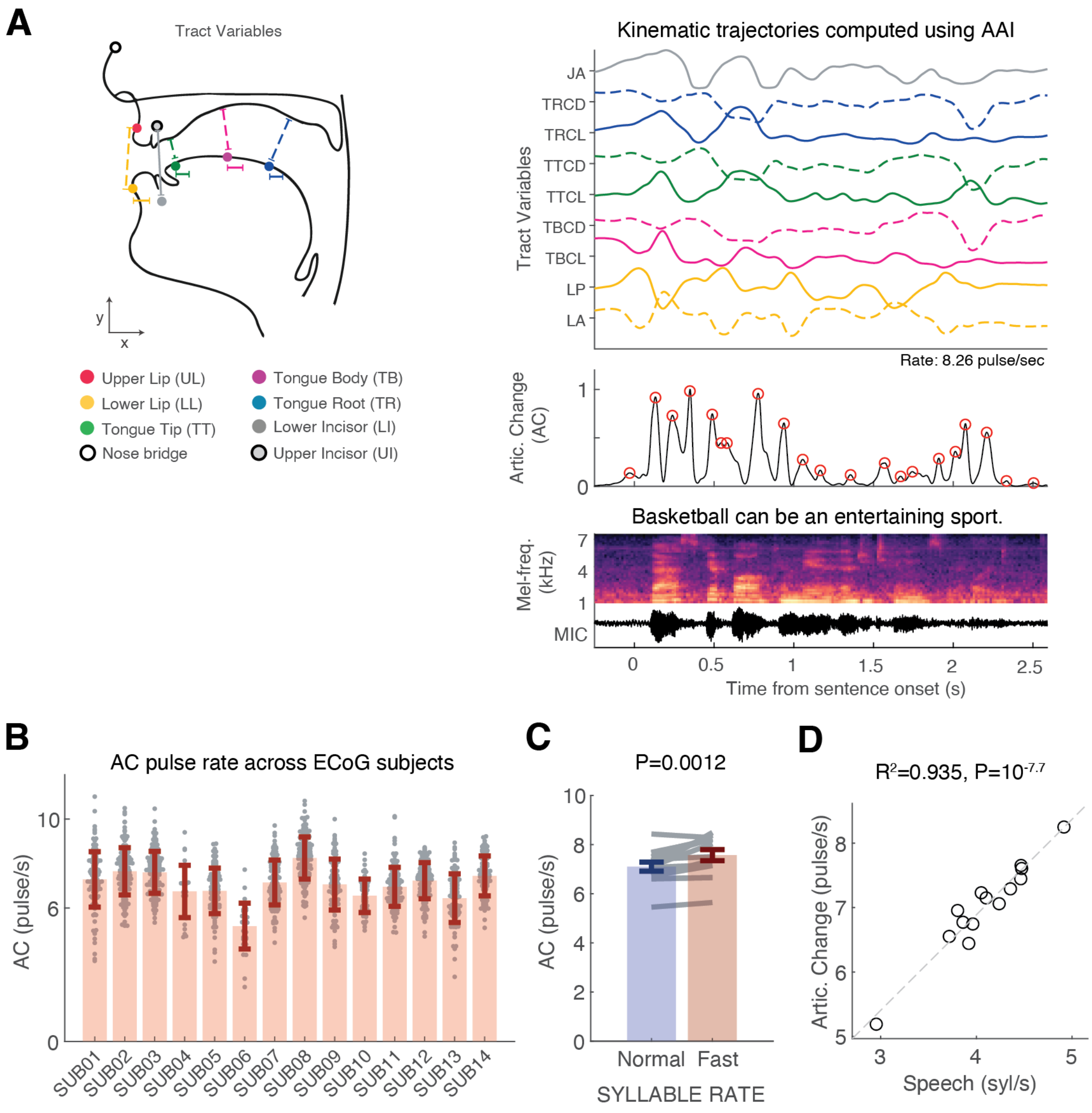

Fig. S7. Articulatory kinematics computed through AAI in ECoG subjects.

(A) Example of articulatory kinematics during continuous speech in a representative ECoG subject. These trajectories were inferred using AAI, based on the produced acoustics. As in actual EMA measurements, articulatory movements exhibited semi-rhythmic modulation, characterized by abrupt, pulse-like changes in the sum of squared velocities across articulators (AC). **(B)** AC pulse rates across 14 ECoG subjects. Each gray dot represents a single sentence; error bars indicate mean ± SD. **(C)** Similar to the pattern observed in the EMA dataset, AC pulse rates in ECoG subjects were higher during faster-spoken sentences ($P<0.01$, Wilcoxon singed-rank test), yet remained centered within the theta range. For normally spoken sentences (10th–90th syllable rate percentiles): mean syllable rate = 4.2 syl/s, AC pulse rate = 7.1 ± 0.18 Hz. For the fastest sentences (>90th percentile): mean syllable rate = 5.5 syl/s, AC pulse rate = 7.6 ± 0.23 Hz). **(D)** Across subjects, AC pulse rate was strongly correlated with the mean syllable rate, highlighting the tight correspondence between these motor events and the resulting speech sounds (R^2=0.93, $P < 10^{-7}$).

bioRxiv preprint doi: https://doi.org/10.1101/2025.10.09.681482; this version posted October 10, 2025. The copyright holder for this preprint (which was not certified by peer review) is the author/funder, who has granted bioRxiv a license to display the preprint in perpetuity. It is made available under aCC-BY 4.0 International license.

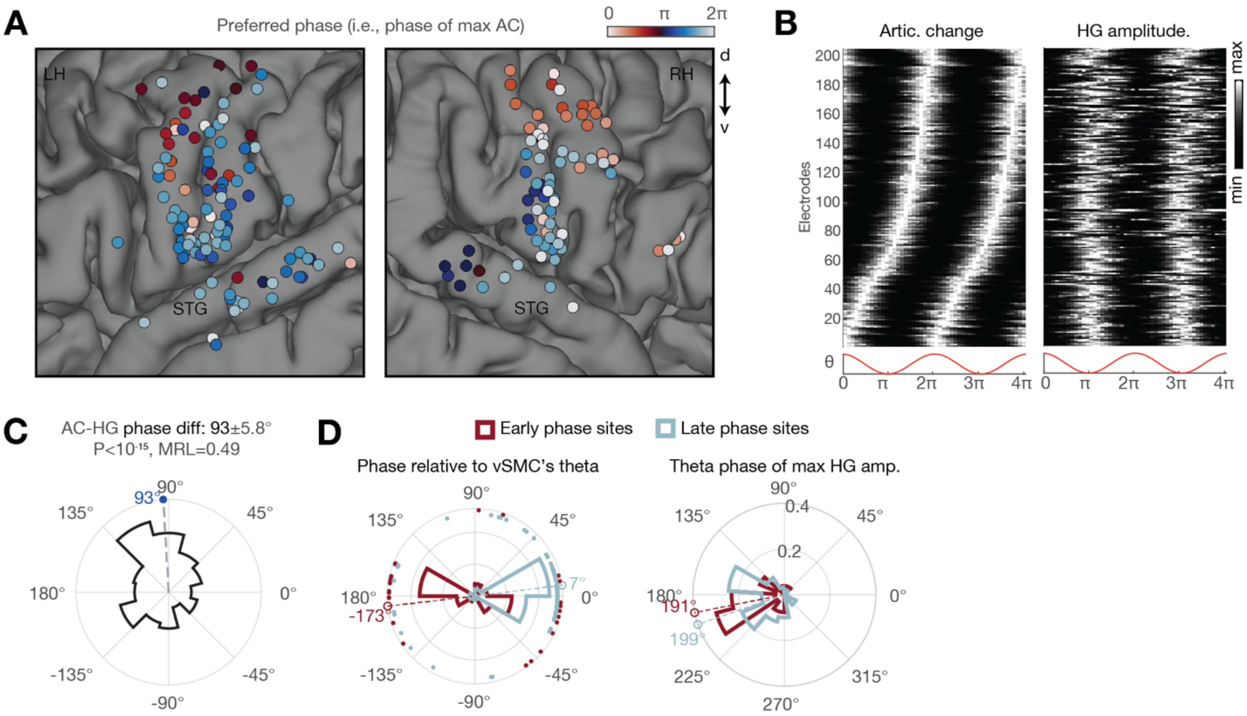

Fig. S8. Consistent phase difference along the SMC dorsoventral axis.

(A) Anatomical distribution of the preferred theta phase (i.e., phase at which AC is maximal) across electrodes with significant coupling (P<0.05, FDR corrected). Notice a cluster of electrodes in the dorsal SMC manifesting coupling to an earlier phase of the theta cycle. **(B)** The electrodes plotted in (A), sorted by their preferred theta phase (phase of maximal AC). Right: the same electrodes, same sorting, but for HG amplitude. Despite variability in the preferred phase for AC, the electrodes exhibit a consistent HG amplitude increase around the theta trough. **(C)** Distribution of the within-electrode difference between theta phase of maximal HG and maximal AC (angular difference: 93° ± 5.8°, mean resultant length |R|=0.49, P<10-15 Rayleigh's test). **(D)** To assess whether the theta rhythm itself was phase-shifted at sites showing early coupling in (B), we calculated the phase difference between each electrode's theta signal and that of the most ventral SMC electrode in each patient, which served as a consistent reference site across subjects (both anatomically and functionally). The analysis revealed that most sites where AC was coupled to an earlier phase of the theta cycle (i.e., before the trough, from ½π to π) exhibited nearly antiphase theta rhythm (-173±15.1°; early-vs-late electrodes: P<0.001) compared to the "late-coupling" more ventral sites (i.e., after the trough, from ³⁄₂π to 2π). Importantly, within each site, the HG amplitude was still consistently coupled to the trough (right panel; early-vs-late electrodes: P=0.52 N.S).

bioRxiv preprint doi: https://doi.org/10.1101/2025.10.09.681482; this version posted October 10, 2025. The copyright holder for this preprint (which was not certified by peer review) is the author/funder, who has granted bioRxiv a license to display the preprint in perpetuity. It is made available under aCC-BY 4.0 International license.

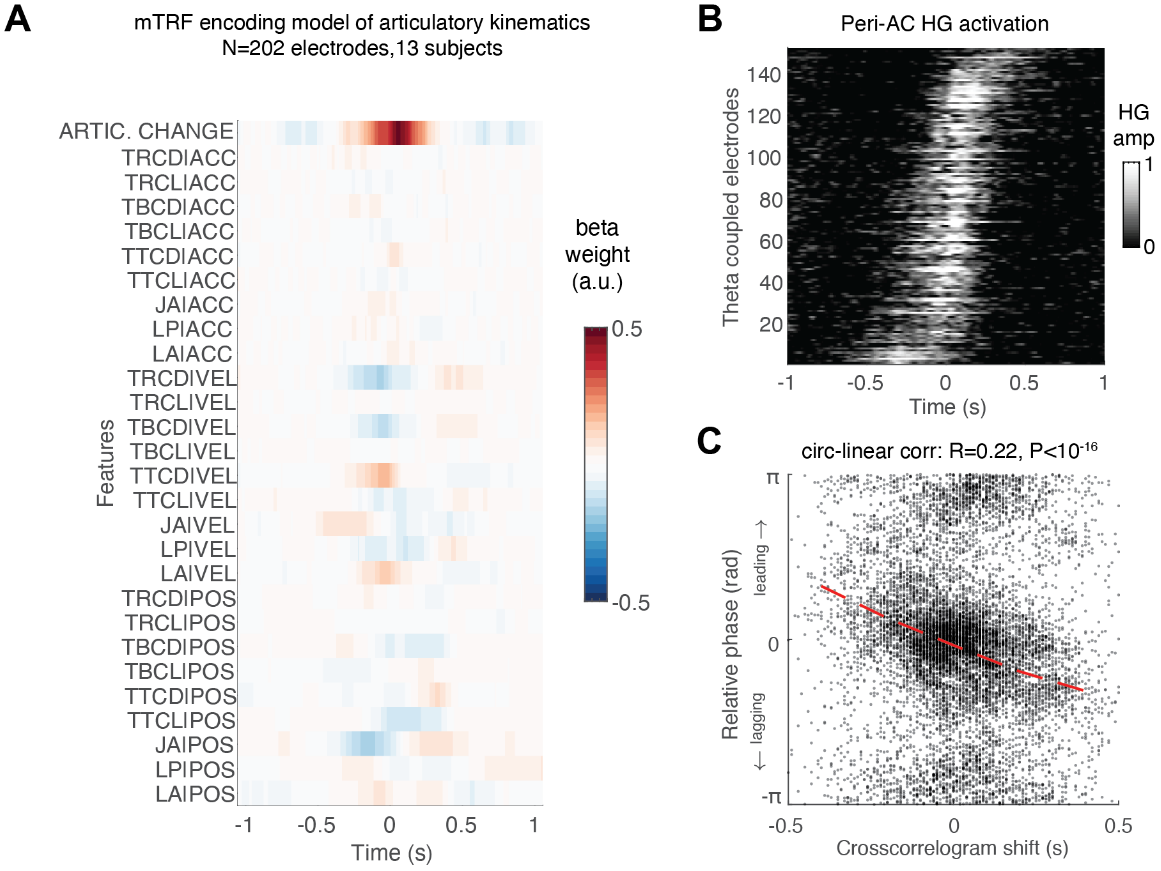

Fig. S9. Mean encoding model beta weights and correlation between peri-AC responses and theta phase.

(A) Beta weights obtained from the mTRF articulatory encoding model, fitted to electrodes exhibiting significant theta-movement coupling ($P < 0.05$, FDR corrected; N = 202 electrodes across 13 subjects). Note the prominent beta weights corresponding to the Articulatory Change feature. This feature stands out among other kinematic features, explaining a significant amount of unique variance in more than 60% of the electrodes ($P < 0.05$, compared to a null distribution generated by circularly shifting the AC feature by a random amount 200 times and refitting the model). Across subjects, this unique variance was significantly greater than zero (proportion of variance: $1 \pm 0.09\%$, $P < 0.0004$, Wilcoxon signed-rank test, N = 14 subjects). **(B)** Across the electrodes exhibiting significant unique variance explained by AC, we observed a progression of response latencies, with some electrodes activating slightly before and others slightly after the AC signal. **(C)** Correlation between the time differences among electrodes—determined by the peaks of the cross-correlograms of peri-AC responses—and their relative theta phase differences (computed in reference to the most ventral SMC site in each subject). We found significant circular-linear correlation across electrode pairs ($R=0.22$, $P<10^{-16}$). Dashed red line, running circular mean.

bioRxiv preprint doi: https://doi.org/10.1101/2025.10.09.681482; this version posted October 10, 2025. The copyright holder for this preprint (which was not certified by peer review) is the author/funder, who has granted bioRxiv a license to display the preprint in perpetuity. It is made available under aCC-BY 4.0 International license.

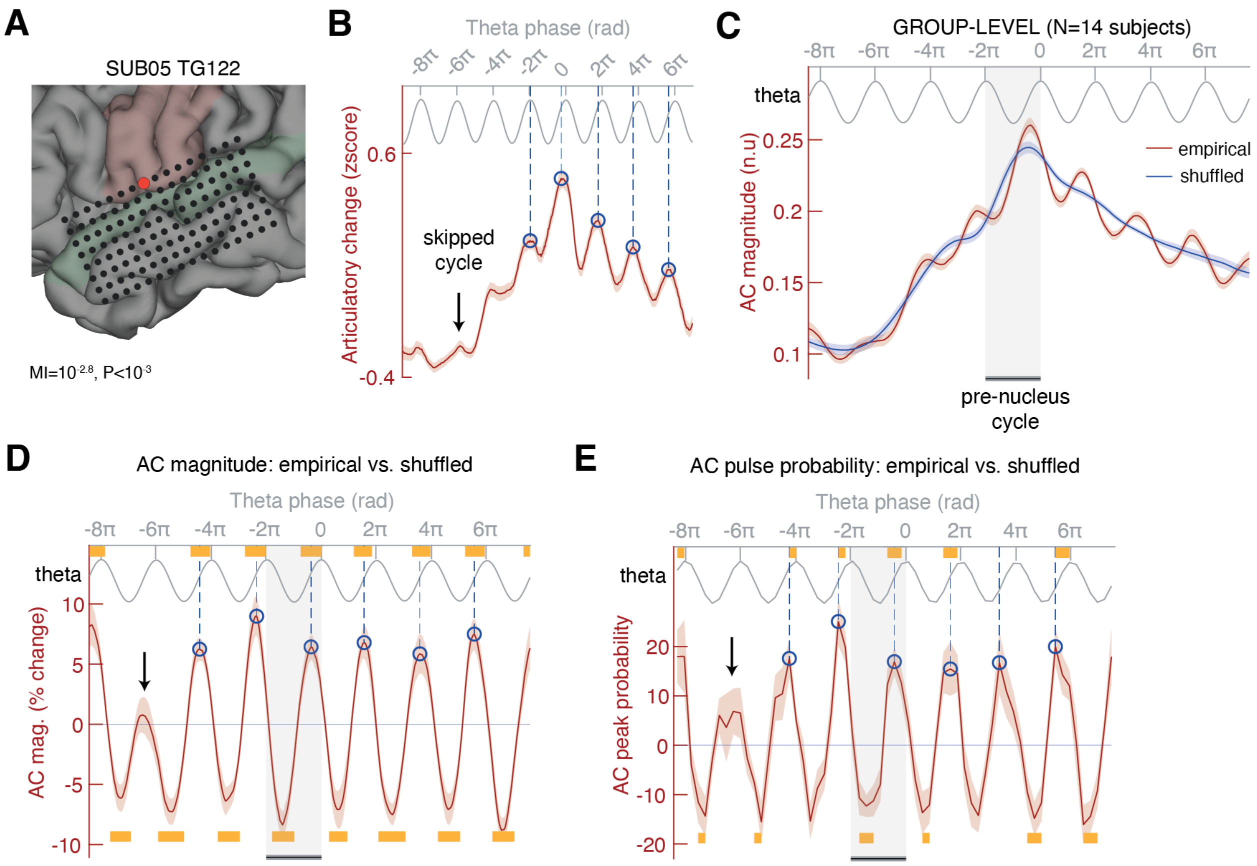

Fig. S10. Theta oscillation coordinates successive vocal-tract movements during syllable production.

(A–B) Same analysis as described in Fig. 6, shown for an electrode from another subject. **(C–E)** Group-level analysis (one electrode per subject, selected for strongest theta–movement coupling) shows that successive AC pulses during syllable production are precisely timed by the ongoing theta rhythm, emerging shortly after the trough and before the subsequent peak. Panel C shows the grand-average AC profile alongside a surrogate profile generated by circularly shifting theta phase in each trial by a random amount (see Methods). Panel D presents AC magnitude expressed as percent change from shuffled data. A cluster-based permutation test revealed significant AC modulation by theta phase (yellow bars indicate statistical significance). The dashed blue line marks the average phase of individual AC peaks, highlighting their consistent alignment with a specific theta phase. Panel E shows a theta phase histogram of AC peaks, displaying the empirical probability distribution normalized by the surrogate distribution. Yellow bars indicate theta phases where the probability of AC pulse was significantly increased/decreased. Shaded areas represent ±SE across subjects. The persistence of this coupling—even after brief pauses in speech (black arrows) during which an entire theta cycle was skipped—further supports the idea that the AC pulses ride on top of the sensorimotor oscillation; that is, the oscillation governs the timing of articulatory movements rather than being driven by them.

bioRxiv preprint doi: https://doi.org/10.1101/2025.10.09.681482; this version posted October 10, 2025. The copyright holder for this preprint (which was not certified by peer review) is the author/funder, who has granted bioRxiv a license to display the preprint in perpetuity. It is made available under aCC-BY 4.0 International license.

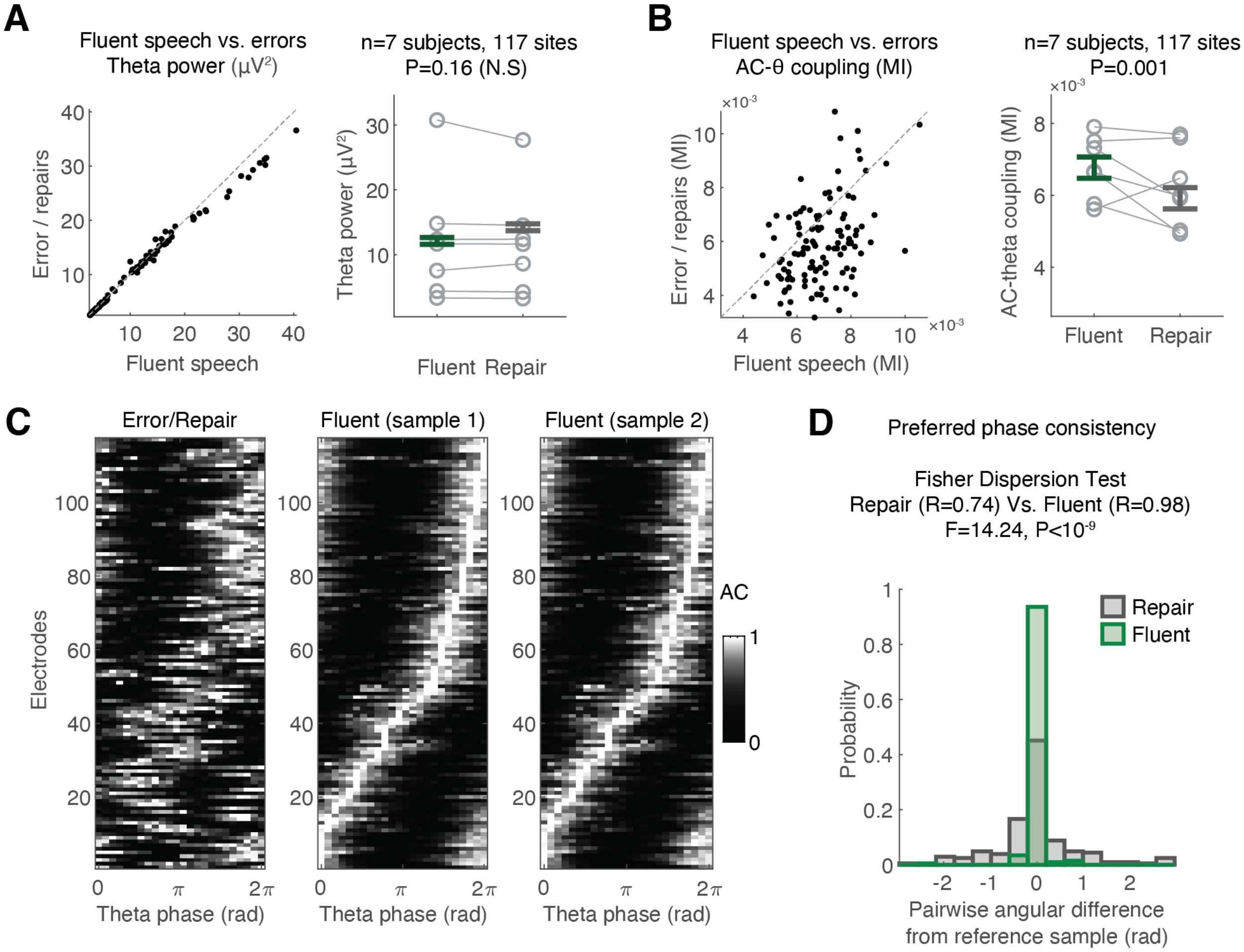

Fig. S11. Disruption of Theta-Movement Coupling During Speech Errors

We compared theta power and theta-movement coupling strength between fluent speech and speech errors/repairs. Subjects with fewer than 10 errors were excluded. To create a comparable fluent speech group with the same number of sentences, we randomly sampled trials while matching syllable rate and sentence duration to the error group. This was repeated 200 times, and median values were used for comparison.

(A) Theta Power: Scatter plot (right) compares theta power between fluent and error trials across electrodes with significant theta-movement coupling. Mixed effects analysis (left) showed no significant difference ($P = 0.16$, mixed effects analysis, 117 sites, 7 subjects). **(B)** Theta-Movement Coupling (MI): Scatter plot (right) compares theta-movement coupling between fluent and error trials. Mixed effects analysis (left) showed a significant difference ($P=0.001$, mixed effects analysis, 117 theta-coupled sites across 7 subjects). **(C)** Preferred phase across electrodes during speech errors/repairs versus fluent sentences (using odd and even samples). Electrodes are sorted based on the even samples (sample 2). The preferred phase remained highly consistent across the two fluent speech samples, whereas speech errors/repairs exhibited a more variable distribution. **(D)** Comparing the (within-electrode-) angular difference between the preferred phase found in odd vs. even samples of fluent speech to the difference found between errors vs. fluent speech revealed greater phase-of-movement variability during trials with errors/repairs (resultant vector length: errors/repairs = 0.73, fluent = 0.99; $P < 10^{-6}$, Fisher dispersion test).

bioRxiv preprint doi: https://doi.org/10.1101/2025.10.09.681482; this version posted October 10, 2025. The copyright holder for this preprint (which was not certified by peer review) is the author/funder, who has granted bioRxiv a license to display the preprint in perpetuity. It is made available under aCC-BY 4.0 International license.

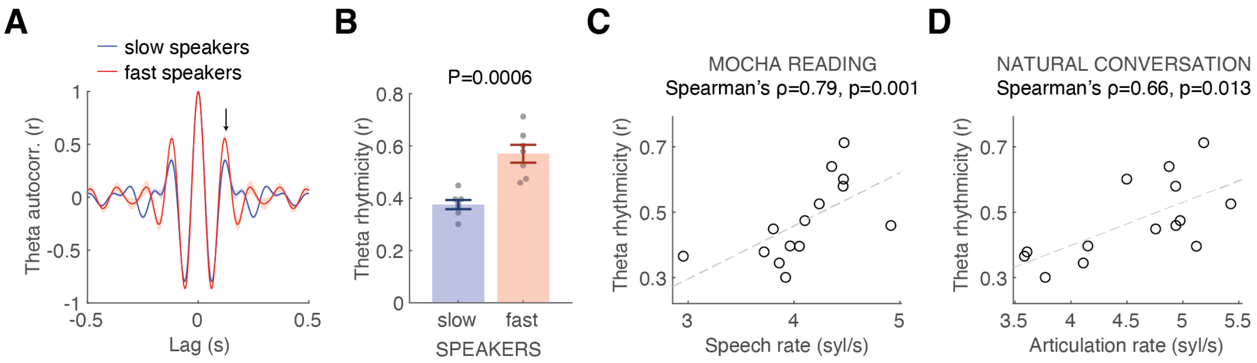

Fig. S12. Faster speech is associated with stronger theta rhythmicity

We analyzed the autocorrelation function (ACF) of the theta-band signal from the top five electrodes showing robust theta–movement coupling. **(A)** We divided the 14 subjects into fast and slow speakers using a median split based on their mean syllable rate. Fast speakers exhibited more pronounced rhythmicity, reflected by a higher secondary peak in the ACF (black arrow). **(B)** Theta rhythmicity was significantly stronger in fast speakers ($P = 0.0006$, rank-sum test). **(C-D)** We observed a significant correlation between median syllable rate and theta rhythmicity both during the MOCHA reading task and during natural conversation. For the latter, we analyzed spontaneous utterances (mean length: 11 ± 3.1 words per utterance) and computed the median articulation rate across utterances (syllables per second, excluding pauses). One subject lacked natural conversation data; for this individual, we used held-out sentence reading data that had not been included in the theta analysis.

bioRxiv preprint doi: https://doi.org/10.1101/2025.10.09.681482; this version posted October 10, 2025. The copyright holder for this preprint (which was not certified by peer review) is the author/funder, who has granted bioRxiv a license to display the preprint in perpetuity. It is made available under aCC-BY 4.0 International license.

References

107. B. Fischl, FreeSurfer. *Neuroimage* **62**, 774–781 (2012).
108. B. D. Argall, Z. S. Saad, M. S. Beauchamp, Simplified intersubject averaging on the cortical surface using SUMA. *Hum Brain Mapp* **27**, 14–27 (2006).
109. Z. S. Saad, R. C. Reynolds, Suma. *Neuroimage* **62**, 768–773 (2012).
110. Y. Norman, O. Raccah, S. Liu, J. Parvizi, R. Malach, Hippocampal ripples and their coordinated dialogue with the default mode network during recent and remote recollection. *Neuron* **109**, 2767-2780.e5 (2021).
111. Y. Norman, E. M. Yeagle, S. Khuvis, M. Harel, A. D. Mehta, R. Malach, Hippocampal sharp-wave ripples linked to visual episodic recollection in humans. *Science (1979)* **365**, eaax1030 (2019).
112. R. S. Desikan, F. Ségonne, B. Fischl, B. T. Quinn, B. C. Dickerson, D. Blacker, R. L. Buckner, A. M. Dale, R. P. Maguire, B. T. Hyman, M. S. Albert, R. J. Killiany, An automated labeling system for subdividing the human cerebral cortex on MRI scans into gyral based regions of interest. *Neuroimage* **31**, 968–980 (2006).
113. A. Wrench, MOCHA: multichannel articulatory database. *MOCHA: multichannel articulatory database.* (1999).
114. J. S. Garofolo, TIMIT: Acoustic-phonetic Continuous Speech Corpus. *(No Title)* (1993).
115. A. Delorme, S. Makeig, EEGLAB: An open source toolbox for analysis of single-trial EEG dynamics including independent component analysis. *J Neurosci Methods* **134**, 9–21 (2004).
116. H. Bokil, P. Andrews, J. E. Kulkarni, S. Mehta, P. P. Mitra, Chronux: A platform for analyzing neural signals. *J Neurosci Methods* **192**, 146–151 (2010).
117. P. Berens, CircStat : A MATLAB Toolbox for Circular Statistics . *J Stat Softw* **31** (2009).
118. B. O. Watson, M. Ding, G. Buzsáki, Temporal coupling of field potentials and action potentials in the neocortex. *European Journal of Neuroscience*, 1–16 (2018).
119. J. Parvizi, S. Kastner, Promises and limitations of human intracranial electroencephalography. *Nat Neurosci* **21**, 474–483 (2018).
120. Y. Nir, L. Fisch, R. Mukamel, H. Gelbard-Sagiv, A. Arieli, I. Fried, R. Malach, Coupling between Neuronal Firing Rate, Gamma LFP, and BOLD fMRI Is Related to Interneuronal Correlations. *Current Biology* **17**, 1275–1285 (2007).
121. Y. Norman, E. M. Yeagle, M. Harel, A. D. Mehta, R. Malach, Neuronal baseline shifts underlying boundary setting during free recall. *Nat Commun* **8** (2017).
122. R. Grandchamp, A. Delorme, Single-trial normalization for event-related spectral decomposition reduces sensitivity to noisy trials. *Front Psychol* **2**, 1–14 (2011).
123. A. Delorme, S. Makeig, EEGLAB: An open source toolbox for analysis of single-trial EEG dynamics including independent component analysis. *J Neurosci Methods* **134**, 9–21 (2004).
124. P. P. Mitra, B. Pesaran, Analysis of Dynamic Brain Imaging Data. *Biophys J* **76**, 691–708 (1999).
125. Y. Norman, E. M. Yeagle, M. Harel, A. D. Mehta, R. Malach, Neuronal baseline shifts underlying boundary setting during free recall. *Nat Commun* **8** (2017).
126. C. J. Cho, P. Wu, T. S. Prabhune, D. Agarwal, G. K. Anumanchipalli, Articulatory Encodec: Vocal Tract Kinematics as a Codec for Speech. 1–14 (2024).
127. N. Seneviratne, G. Sivaraman, C. Espy-Wilson, Multi-corpus acoustic-to-articulatory speech inversion. *Proceedings of the Annual Conference of the International Speech Communication Association, INTERSPEECH* **2019-Septe**, 859–863 (2019).
128. M. Tiede, C. Y. Espy-Wilson, D. Goldenberg, V. Mitra, H. Nam, G. Sivaraman, Quantifying kinematic aspects of reduction in a contrasting rate production task. *J Acoust Soc Am* **141**, 3580–3580 (2017).
129. G. K. Anumanchipalli, J. Chartier, E. F. Chang, Speech synthesis from neural decoding of spoken sentences. *Nature* **568**, 493–498 (2019).
130. G. K. Anumanchipalli, K. Prahallad, A. W. Black, Festvox : Tools for Creation and Analyses of Large Speech Corpora. *Workshop on very large scale speech processing*, 2–3 (2011).
131. M. J. Crosse, N. J. Zuk, G. M. Di Liberto, A. R. Nidiffer, S. Molholm, E. C. Lalor, Linear Modeling of Neurophysiological Responses to Speech and Other Continuous Stimuli: Methodological Considerations for Applied Research. *Front Neurosci* **15** (2021).
132. C. R. Holdgraf, J. W. Rieger, C. Micheli, S. Martin, R. T. Knight, F. E. Theunissen, Encoding and decoding models in cognitive electrophysiology. *Front Syst Neurosci* **11**, 61 (2017).
133. D. M. Groppe, T. P. Urbach, M. Kutas, Mass univariate analysis of event-related brain potentials/fields I: A critical tutorial review. *Psychophysiology* **48**, 1711–1725 (2011).
134. E. Maris, R. Oostenveld, Nonparametric statistical testing of EEG- and MEG-data. *J Neurosci Methods* **164**, 177–190 (2007).

bioRxiv preprint doi: https://doi.org/10.1101/2025.10.09.681482; this version posted October 10, 2025. The copyright holder for this preprint (which was not certified by peer review) is the author/funder, who has granted bioRxiv a license to display the preprint in perpetuity. It is made available under aCC-BY 4.0 International license.

135. OpenWetWare, Mass Univariate ERP Toolbox: Other EEG Analysis Software Packages --- OpenWetWare{,}. [Preprint] (2015).
136. Y. Benjamini, D. Yekutieli, The control of the false discovery rate in multiple testing under dependency. *Ann Stat* **29**, 1165–1188 (2001).
